# CuAAC-Derived Selective Fluorescent Probe as a Recognition Agent for Pb(II) and Hg(II): DFT and Docking Studies

**DOI:** 10.1021/acsomega.2c05050

**Published:** 2022-10-20

**Authors:** Gurleen Singh, Nancy George, Riddima Singh, Gurjaspreet Singh, Jashan Deep Kaur, Gurpreet Kaur, Harminder Singh, Jandeep Singh

**Affiliations:** † School of Chemical Engineering and Physical Sciences, Lovely Professional University, Phagwara, Punjab 144411, India; ‡ Department of Chemistry and Centre of Advanced Studies in Chemistry, Panjab University, Chandigarh 160014, India; § Department of Chemistry, GGN Khalsa College, Ludhiana, Punjab 141001, India

## Abstract

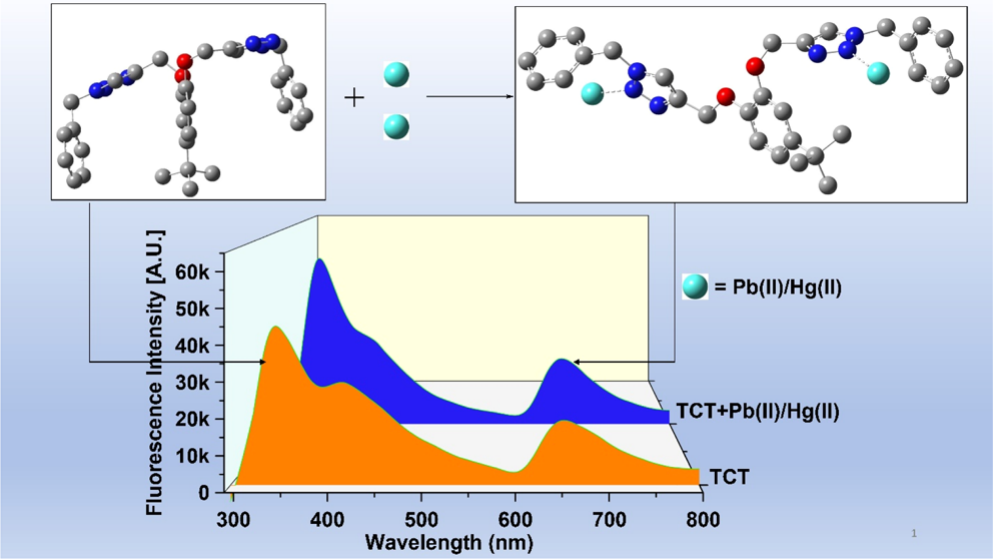

Copper(I)-catalyzed alkyne–azide cycloaddition (CuAAC) is a resourceful and stereospecific methodology that has considerably yielded promising 1,2,3-triazole-appended “click” scaffolds with the potential for selective metal ion recognition. Based on “click” methodology, this report presents a chemosensor probe (TCT) based on 4-*tert*-butylcatechol architecture, via the CuAAC pathway, as a selective and efficient sensor for Pb(II) and Hg(II) ions, categorized as the most toxic and alarming environmental contaminants among the heavy metal ions. The synthesized probe was successfully characterized by spectroscopy [IR and NMR (^1^H and ^13^C)] and mass spectrometry. The chemosensing study performed in acetonitrile/water (4:1) solvent media, via UV–vis and fluorescence spectroscopy, established its selective sensitivity for Pb(II) and Hg(II) species among the list of explored metal ions with the limits of detection being 8.6 and 11 μM, respectively. Additionally, the ^1^H NMR and IR spectra of the synthesized TCT–metal complex also confirmed the metal–ligand binding. Besides, the effect of time and temperature on the binding ability of TCT with Pb(II) and Hg(II) was also studied via UV–vis spectroscopy. Furthermore, density functional theory studies put forward the structural comprehension of the sensor by availing the hybrid density functional (B3LYP)/6311G++(d,p) basis set of theory which was subsequently utilized for investigating its anti-inflammatory potential by performing docking analysis with human leukotriene b4 protein.

## 1 Introduction

The consistent search for efficient and suitable scaffolds for toxic heavy metal ion(s) recognition has been the core idea of ongoing research among the various researchers worldwide owing to their potential deleterious effects on humans and their ecotoxicological presence in the environment.^1−3^ The presence of toxic ions such as Pb(II), Hg(II), and so forth above the permissible threshold value can alter the biomolecules mainly via oxidative damage and diminishing of enzymatic activities,^4,5^ thereby interfering with the vital processes occurring inside the body to sustain life. Lead has become a ubiquitous heavy metal globally.^6^ On accumulation in significant quantities in the soft tissues such as the brain, kidneys, heart, and so forth, it can interact with the physiologically active groups of different proteins and hence incapacitate their biological functions, which result in serious health hazards such as hematological effects (anemia), nephrotoxicity (interstitial nephritis), cardiovascular effects (hypertension), neurological effects (lead encephalopathy), and so forth.^7−11^ Mercury in its inorganic form [Hg(II)] is severely toxic to living organisms, and the startling consideration that other forms of mercury are convertible to the insidiously toxic divalent form via “biomethylation” keeps the researchers on their toes for controlling mercury accretion in the environment.^12^ Significant concentrations of mercury in the body impairs enzymatic activity and causes mitochondrial dysfunction and increased oxidative stress, thereby resulting in complications such as hypertension, cardiac arrhythmia, coronary heart disease, atherosclerosis, and so forth.^12−16^ Therefore, a constant need to confirm the presence of such toxic and non-biodegradable heavy metal ions in the environment becomes indispensable to keep their accumulation in the environment well within the permissible limits.

**Figure 1:**
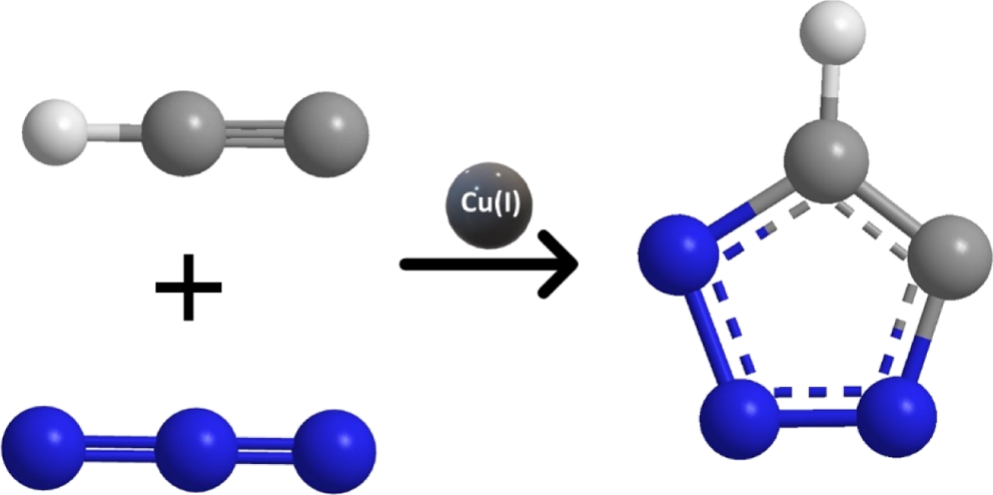
Illustrative portrayal of CuAAC pathway to form 1,2,3-triazole.

The archetypal click reaction, that is, CuAAC^17^ methodology, provides a well-organized, robust, and efficient pathway to synthesize 1,2,3-triazole-based compounds for addressing the research problem of metal ion toxicity in addition to other sensing devices such as supramolecular polymers.^18−20^ The development of new 1,2,3-triazole-linked compounds by stitching a terminal alkyne to an organic azide in the presence of Cu(I) catalyst^21^ (Figure 1) has been extensively explored over the past several years with increasing interest as ion-recognition devices^22−25^ due to their high sensitivity, rapid response time, lower toxicity, strong biological activity, and a high degree of environmental compatibility as well as their coordination abilities,^26^ as the heteroatom-bearing aromatic rings exhibit binding to specific metal ions via ion–dipole interactions.^27^ Besides, the flexibility provided by this methodology to insert tailor-made functionalities to the 1,2,3-triazole moiety empowers the researchers to develop recognition devices that provide distinct advantages such as lower limit of detection (LoD) and visual changes to the naked eye over the other existing methodologies for ion recognition such as atomic absorption spectroscopy^28^ and inductively coupled plasma–mass spectrometry.^29^

In the present work, we report the design and synthesis of a novel 1,2,3-triazole-tethered 4-*tert*-butylcatechol-based chemosensor probe (TCT), synthesized via the CuAAC “click” pathway, that was explored as a selective sensor for the highly toxic Pb(II) and Hg(II) ion recognition via UV–vis and fluorescence spectroscopies. The chemosensing potential of the probe was evident from the fluctuations in its absorption and emission spectra on addition of the above-mentioned metal ions in CH_3_CN/H_2_O (4:1) solvent media. Above and beyond, the probe was structurally optimized via density functional theory (DFT) using the hybrid density functional (B3LYP)/6311G++(d, p) basis set of theory and subsequently docked with human leukotriene b4 to ascertain its anti-inflammatory effects. Although the literature is abundant with 1,2,3-triazole-based chemosensors for Pb(II) and Hg(II) sensing either distinctly^30−33^ or in addition to some other metal ions such as Fe^3+^, Cu^2+^, Zn^2+^, and so forth,^34−37^ this is the first report, to the best of our knowledge, wherein a single 1,2,3-triazole-appended ligand is a potential recognition agent for concomitant sensing of Pb(II) and Hg(II).

## 2 Synthesis

**Caution!** Azide compounds are heat and shock sensitive. Great care and protection are required for handling them.

### 2.1 Materials and Method

All the syntheses were done under normal laboratory conditions. Starting materials 4-*tert*-butylcatechol (LOBA), propargyl bromide solution (80% by weight in toluene) (Spectrochem), *N*,*N*-dimethylformamide (DMF) (LOBA), cesium carbonate (LOBA), tetrahydrofuran (THF) (LOBA), triethylamine (Et_3_N) (SDFCL), and bromotris(triphenylphosphine)copper(I) [CuBr(PPh_3_)_3_] (Aldrich) were used as received. The synthesized compounds were characterized via different spectroscopic techniques, namely, IR spectra (neat) using a SHIMADZU FTIR-8400S spectrometer and multinuclear NMR (^1^H, ^13^C) spectra on a Bruker Advance Neo FT NMR spectrometer. CDCl_3_ was used in NMR (^1^H and ^13^C) as an internal reference, and the chemical shifts reported were relative to tetramethylsilane (TMS). Mass spectra (LCMS) were recorded on a Bruker make mass spectrometer model Esquire 3000. The melting points were uncorrected and determined in sealed capillary tubes using a Mel Temp II device. CHN analyses were attained on a PerkinElmer model 2400 CHNS elemental analyzer. Ion sensing studies were done using a SHIMADZU UV-1900 spectrometer and a PerkinElmer FL 6500 fluorescence spectrophotometer using quartz cuvettes. Theoretical scrutiny was undertaken using DFT, with the hybrid density functional (B3LYP)/6311G++(d,p) basis set of theory. Docking studies were performed via AutoDock Vina.

### 2.2 Synthesis of 4-*tert*-Butylcatechol Alkyne (2)

**Scheme 1:**
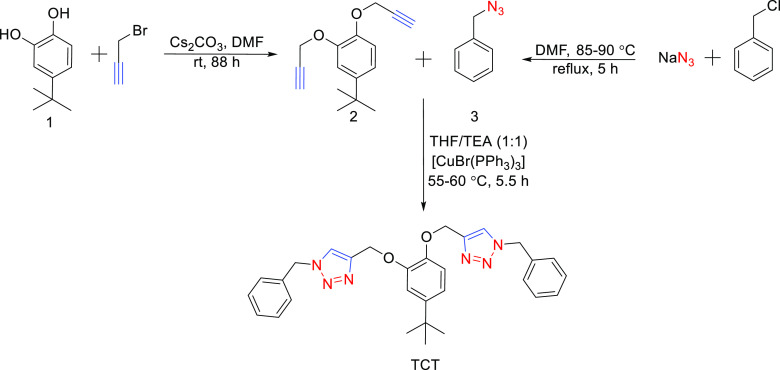
Schematic Illustration of the Reaction Route to Convert 4-*tert*-Butylcatechol into its Corresponding Alkyne (2), Followed by Synthesis of 1,2,3-Triazole-Based Probe (TCT) via a Combination of 2 with Benzyl Azide (3)

4-*tert*-Butylcatechol (1) (1.0 g, 6.0 mmol, 1 equiv) was dissolved in DMF (15.0 mL) under continuous stirring. To this solution, anhydrous cesium carbonate (6.84 g, 21.0 mmol, 3.5 equiv) was added with subsequent dropwise addition of propargyl bromide (2.05 g, 13.8 mmol, 2.3 equiv) within 10 min, the reaction mixture was stirred at room temperature, and the progress of the reaction was monitored by TLC (ethyl acetate/hexane, 1:4), which was completed within 88 h. The reaction was quenched by the addition of ice-cold water, and the product was extracted with ethyl acetate. The combined organic layers were dried over anhydrous sodium sulfate, filtered, and subjected to vacuum evaporation for solvent elimination (Scheme 1).

#### 2.2.1 4-*tert*-Butylcatechol Alkyne (2) (4-(*tert*-Butyl)-1,2-bis(prop-2-yn-1-yloxy)benzene)

Yield: 95%; color/texture: dark brown viscous oil; MF: C_16_H_18_O_2_; Elem. Anal. Calcd (%): C = 79.31, H = 7.49, O = 13.20, Found (%): C, 79.35; H, 7.47; O, 13.18; IR (neat, cm^–1^): 3287, 2959, 2868, 2121, 1755, 1591, 1504, 1455, 1414, 1366, 1261, 1198, 1143, 1110, 1014, 926, 854, 636; ^1^H NMR (500 MHz, CDCl_3_): δ = 7.11 (s, 1H), 6.96 (d, *J* = 1.0 Hz, 2H), 4.74 (d, *J* = 2.4 Hz, 2H), 4.70 (d, *J* = 2.4 Hz, 2H), 2.49 (dt, *J* = 6.9, 2.3 Hz, 2H), 1.30 (s, 9H); ^13^C NMR (126 MHz, CDCl_3_): δ = 146.99 (s), 145.54 (s), 145.35 (s), 118.78 (s), 114.55 (s), 113.67 (s), 78.91 (s), 75.75 (d), 57.20 (s), 56.95 (s), 34.41 (s), 31.44 (s).

### 2.3 Synthesis of Benzyl Azide (3)

The starting material benzyl chloride (5.5 g, 47.8 mmol, 1 equiv) was dissolved in DMF (25 mL), and sodium azide (15.5 g, 238.9 mmol, 5 equiv) was added to it. The reaction mixture was refluxed at 85–90 °C for 5 h, and the product was extracted with ethyl acetate. The combined organic layers were dried over anhydrous sodium sulfate, filtered, and subjected to vacuum evaporation for solvent elimination (Scheme 1).

#### 2.3.1 Benzyl Azide (3)

Yield: 60%; color/texture: light yellow oil; MF: C_7_H_7_N_3_; Elem. Anal. Calcd (%): C, 63.14; H, 5.30; N, 31.56; Found (%): C, 63.12; H, 5.31; N, 31.57; IR (neat, cm^–1^): 3032, 2930, 2090, 1495, 1452, 1347, 1253, 1201, 876, 740, 697, 568, 463; ^1^H NMR (500 MHz, CDCl_3_): δ = 7.27–7.12 (m, 5H), 4.14 (s, 2H); ^13^C NMR (126 MHz, CDCl_3_): δ = 135.53 (s), 128.91 (s), 128.37 (s), 128.31 (s), 54.82 (s).

### 2.4 Synthesis of 4-*tert*-Butylcatechol-Based Triazole (TCT)

4-*tert*-Butylcatechol alkyne (2) (0.7 g, 2.9 mM, 1 equiv) was dissolved in THF/Et_3_N (1:1) solution. Then, benzyl azide (0.77 g, 5.8 mM, 1 equiv) was added to the reaction mixture, followed by the addition of Cu (I) catalyst (0.001 mmol). The reaction mixture was refluxed at 55–60 °C for 5 h. The completion of the reaction was determined via TLC (ethyl acetate/hexane, 1:4). The reaction was quenched with ice-cold water, and the solid product was filtered, washed with water (3× 5 mL), and then dried (Scheme 1).

#### 2.4.1 4-*tert*-Butylcatechol Triazole (TCT) (4,4′-(((4-(*tert*-Butyl)-1,2-phenylene)bis(oxy))bis(methylene))bis(1-Benzyl-1*H*-1,2,3-Triazole))

Yield: 81%; color/texture: light brown fine powder; mp 120–122 °C; MF: C_30_H_32_N_6_O_2_; Elem. Anal. Calcd (%): C, 70.84; H, 6.34; N, 16.52; O, 6.29; Found (%): C, 70.88; H, 6.31; N, 16.49; O, 6.31; IR (neat, cm^–1^): 3134, 3062, 2956, 1591, 1511, 1460, 1384, 1315, 1255, 1200, 1145, 1013, 800, 694; ^1^H NMR (500 MHz, CDCl_3_): δ = 7.58 (d, *J* = 8.3 Hz, 2H), 7.32 (dt, *J* = 4.3, 2.2 Hz, 6H), 7.22 (td, *J* = 6.8, 3.0 Hz, 4H), 7.04 (d, *J* = 2.0 Hz, 1H), 6.98–6.87 (m, 1H), 5.45 (s, 4H), 5.20 (s, 2H), 5.16 (s, 2H), 1.24 (s, 9H); ^13^C NMR (126 MHz, CDCl_3_): δ = 147.85 (s), 146.40 (s), 145.49 (s), 134.67 (s), 129.07 (s), 128.69 (s), 128.10 (s), 128.07 (s), 123.12 (s), 123.09 (s), 118.86 (s), 115.09 (s), 114.03 (s), 63.91 (s), 63.67 (s), 54.09 (s), 34.39 (s), 31.44 (s), LC–MS: *m*/*z* (actual) = 508.63; *m*/*z* (experimental) = 509.50 (M + 1).

## 3 Results and Discussion

### 3.1 Synthesis

The 4-*tert*-butylcatechol-linked 1,2,3-triazole derivative (TCT) was synthesized via a two-step sequential pathway in which initially, the starting material, that is, 4-*tert*-butylcatechol, was subjected to nucleophilic substitution with propargyl bromide resulting in the substitution of two labile protons of the −OH groups of the former with propargyl groups to produce the corresponding alkyne (2), which was further reacted with benzyl azide (3) in the presence of THF/TEA (1:1) solvent system and [CuBr(PPh_3_)_3_] as the catalyst in a cycloaddition step to yield the corresponding triazole TCT. The azide was separately synthesized via the procedure reported by Ferreira et al.^38^ The product and its precursor alkyne were characterized using common spectroscopic techniques (FTIR, ^1^H NMR, and ^13^C NMR) and mass spectrometry and further applied for ion sensing purposes.

### 3.2 Spectroscopic Analysis

#### 3.2.1 IR Spectroscopy

The data obtained for terminal alkyne (2), benzyl azide (3), and TCT via IR spectroscopy in the range of 4000–500 cm^–1^ were in accordance with the expected results. The peaks observed in the IR spectrum of the alkyne (2) at 3287 and 2121 cm^–1^ corresponded to ≡C–H str. and C≡C str., respectively. The IR spectrum of azide (3) confirmed the presence of −N_3_ group by displaying an intense peak at 2090 cm^–1^. The disappearance of peaks at 3287, 2121, and 2090 cm^–1^ in the IR spectra of TCT specified the convergence of alkyne and azide moieties to yield the 1,2,3-triazole moiety. Additionally, the peak at 3134 cm^–1^ in the IR spectra of TCT paralleled the −C=C–H str. of the triazole ring.

#### 3.2.2 NMR Spectroscopy and Mass Spectrometry

The successful synthesis of the alkyne (2) and TCT was confirmed via NMR spectra (^1^H and ^13^C). The doublet of triplet at δ = 2.49 ppm indicative of the alkynyl (≡C–H) protons in the ^1^H NMR spectrum of the alkyne (2) was missing in the ^1^H NMR spectrum of TCT, thereby confirming its conversion to form the 1,2,3-triazole moiety during the cycloaddition reaction of alkyne and azide. Consequently, the signal due to the alkynyl proton appeared in the aromatic region in the ^1^H NMR spectrum of TCT. Additionally, the −CH_2_–O– protons of the alkyne (2) were then present in the vicinity of the aromatic 1,2,3-triazole ring in TCT, which was testified by the downfield shift of peak at δ = 4.79 ppm in the ^1^H NMR spectrum of the alkyne (2). Peaks at δ = 75.7 ppm and δ = 78.9 ppm suggestive of C≡C moiety in the ^13^C NMR spectrum of the alkyne (2) were missing in the ^13^C NMR of triazole (4), thereby testifying to their modification to get embedded into the 1,2,3-triazole ring. The molar mass observed at *m*/*z* = 509.50 for TCT confirmed its successful formation. All the spectral records have been provided in the Supporting Information.

### 3.3 UV–Vis Analysis

**Figure 2:**
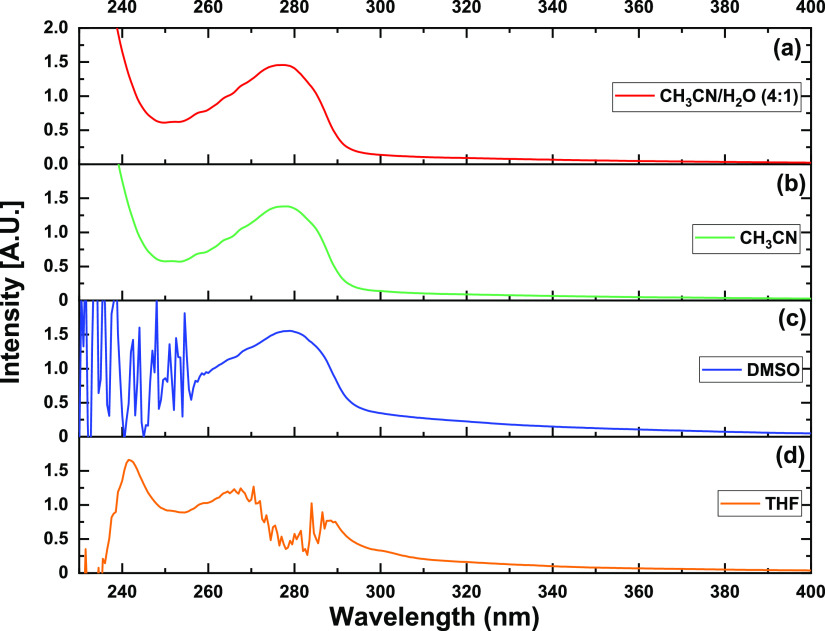
Absorption spectrum of 0.4 mM solution of TCT in different solvent media: (a) CH_3_CN/H_2_O, (b) CH_3_CN, (c) DMSO, and (d) THF.

**Figure 3:**
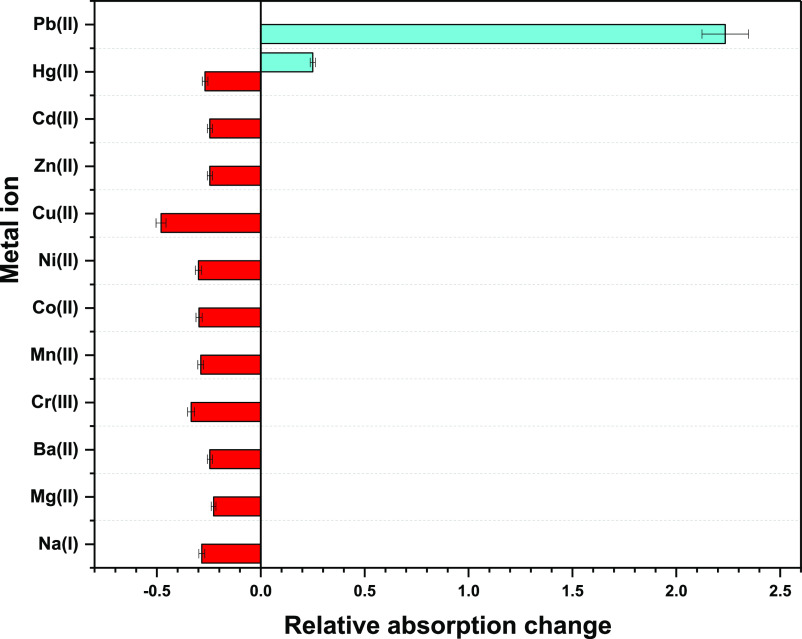
Relative change observed in absorption behavior of TCT with different metal ions in CH_3_CN/H_2_O (4:1) solvent media on successive addition of 15 equiv of every metal ion solution.

The 1,2,3-triazole-appended probe TCT exhibited copasetic spectral results for sensing purposes. The solvatochromic analysis of TCT led to the selection of a mixture of acetonitrile and water (4:1) as the solvent media of choice for UV–vis studies among DMSO, THF, acetonitrile, and acetonitrile/water (4:1) owing to its solubility and fine spectral results, as shown in Figure 2. The solution of TCT was set to a concentration of 0.4 mM after optimization of solution concentration for UV–vis studies. The probe exhibited an absorption maximum at 277 nm corresponding to an intensity of 1.5 (Supporting Information, Figure S11). The metal ion solutions of 1 mM concentration of Cr(III), Mn(II), Co(II), Ni(II), Cu(II), Zn(II), Cd(II), Hg(II), Pb(II), Na(I), Mg(II), and Ba(II) were prepared in acetonitrile/water (4:1) and subsequently tested for sensing studies with TCT wherein only the metal ion solutions of Pb(II) and Hg(II) (1 mM) were observed to successfully induce significant fluctuations in the absorption spectrum of TCT (Figure 3).

#### 3.3.1 Response of 1,2,3-Triazole Probe toward Pb(II) and Hg(II)

**Figure 4:**
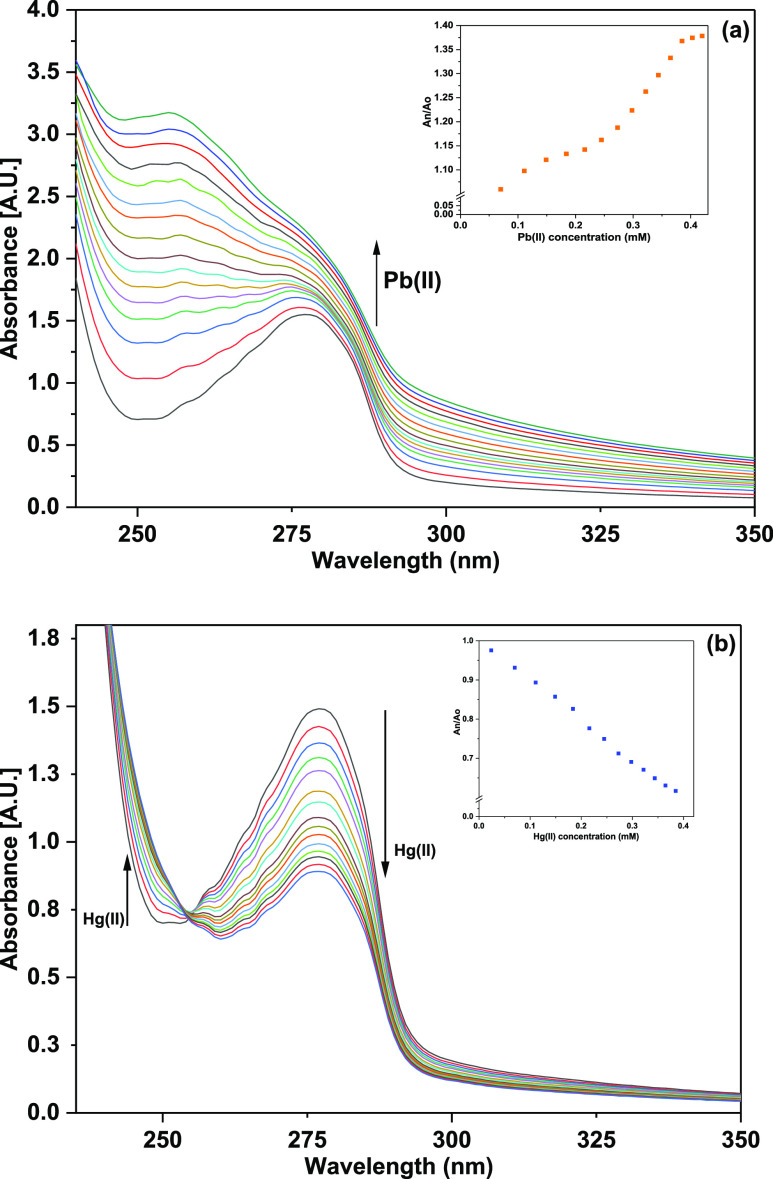
UV–vis titration spectra of TCT on incremental addition of 15 equiv of 1 mM solution of (a) Pb(II) and (b) Hg(II) in CH_3_CN/H_2_O (4:1); the inset exhibits the relative absorbance of TCT (*A*_n_/*A*_o_) vs molar concentration of the metal ion (mM).

The ion detection potential of TCT with Pb(II) and Hg(II) ions was explored via UV–vis spectral analysis by titrating 0.4 mM TCT solution with 15 equiv of 1 mM each of Pb(II) and Hg(II) solutions as illustrated in Figure 4a,b, respectively. During the titrations, the probe concentration was kept constant at 0.4 mM, while the metal ion concentration was sequentially increased from 0 to 15 equiv. When Pb(II) ions were gradually added to the ligand solution, TCT exhibited an intense hyperchromic shift accompanied by a blue shift of about 22 nm as shown in Figure 4a, thereby confirming the binding of Pb(II) to the probe solution. The inset of relative change in absorbance maxima (*A*_n_/*A*_o_) versus the molar concentration of Pb(II) for peak at 277 nm upon successive addition of 15 equiv of Pb(II) ions is also embedded in Figure 4a, where *A*_n_ = absorbance maxima on incremental addition of Pb(II) ions and *A*_o_ = absorbance maxima of the probe. In the case of titration with Hg(II) ions, TCT displayed a gradual hypochromic shift in the absorption intensity at 277 nm with an isosbestic point appearing at 255 nm (Figure 4b). The inset shows the relative change in absorbance maxima (*A*_n_/*A*_o_) versus the molar concentration of metal ion solution for peak at 277 nm upon successive addition of 15 equiv of Pb(II) ions, where *A*_n_ = absorbance maxima on incremental addition of Pb(II) ions and *A*_o_ = absorbance maxima of the probe.

#### 3.3.2 Competitive Metal Ion Titrations

The practical utility of the probe to selectively sense Hg(II) regardless of the presence of other metal ions was confirmed by carrying out competitive ion titration of 0.4 mM probe solution [in CH_3_CN/H_2_O (4:1)] with a solution comprising an equimolar concentration of different metal ions. The results obtained from the absorption spectrum (Supporting Information, Figure S12) after the titrations suggested that the Hg(II) identification capability of the sensor probe remained unaffected even in the presence of other metal ions. Furthermore, the probe was also investigated, via UV–vis spectroscopy, for its preference to selectively sense either Pb(II) or Hg(II), when both the metal ions were present in the solution. Titrating the probe solution with the equimolar solution of Pb(II) and Hg(II) ions displayed an absorption spectrum similar to that obtained for Hg(II), thereby confirming the higher selectivity of the probe for Hg(II) over Pb(II) (Supporting Information, Figure S13).

#### 3.3.3 Time-Dependent Study of the Metal–Ligand Complex

The effect of time on the binding of TCT with Pb(II) as well as Hg(II) ions was analyzed for 1 h, causing the absorption changes on complexes TCT–Pb(II) and TCT–Hg(II). The spectral results are presented in the Supporting Information (Figure S14), which suggest that the absorption intensity of the TCT–Pb(II) complex witnessed a gradual decrease over time, whereas the absorption intensity of the TCT–Hg(II) complex exhibited an increase in the absorption intensity toward the latter part of the spectrum. Besides, the addition of the aforementioned metal ions to the probe solution exhibited instantaneous absorption and fluorescence intensity changes, thereby indicating that TCT is a proficient “no-wait” sensor for Pb(II) and Hg(II).

#### 3.3.4 Temperature Dependence of Metal–Ligand Binding

The applicability of the probe for metal ion recognition was also tested over a temperature range. The metal–ligand complex solutions of TCT–Pb(II) and TCT–Hg(II) were subjected to a temperature range between 30 and 50 °C, and their corresponding absorption spectra were recorded at a difference of 2 °C. The spectral results have been compiled and presented in the Supporting Information (Figure S15), wherein it is observed that for the TCT–Pb(II) complex, the absorption intensity decreases with the decrease in temperature. However, the time dependence has also to be taken into consideration, which is the reason behind the observed diminishing absorption intensity with respect to temperature. The same can be confirmed by observing the absorption intensity values of the TCT–Pb(II) complex at 36 and 34 °C, which were recorded after a relatively extra time lag as compared to other values. In the case of the TCT–Hg(II) complex, the represented spectra exhibited a gradual increase in absorption intensity with decreasing temperature, which could also be attributed to the time-dependent behavior of the probe instead of the temperature effect. Therefore, it was concluded that the binding of the probe with both Pb(II) and Hg(II) was independent of temperature.

### 3.4 Fluorescence Studies

**Figure 5:**
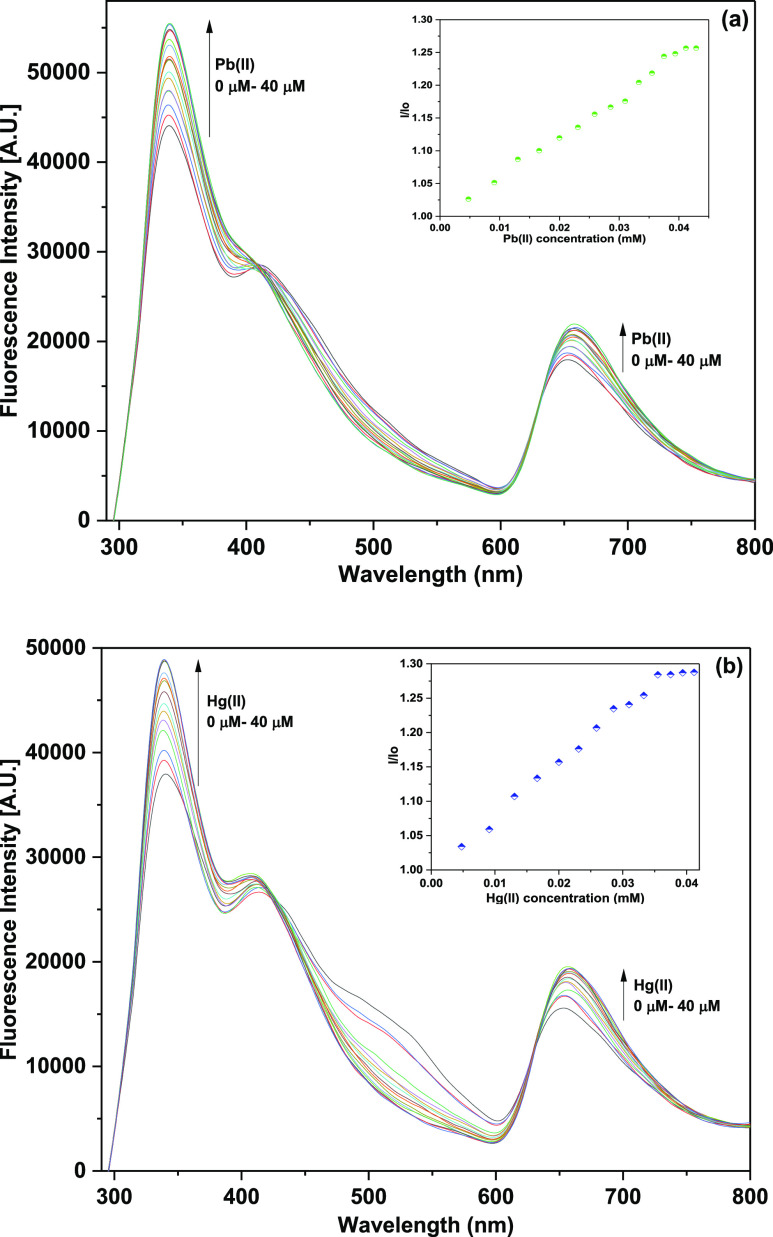
Observed enhancement in fluorescence emission of probe TCT on incremental addition of (a) Pb(II) ions and (b) Hg(II) ions in CH_3_CN/H_2_O (4:1); the inset shows the relative emission change vs metal ion concentration.

**Figure 6:**
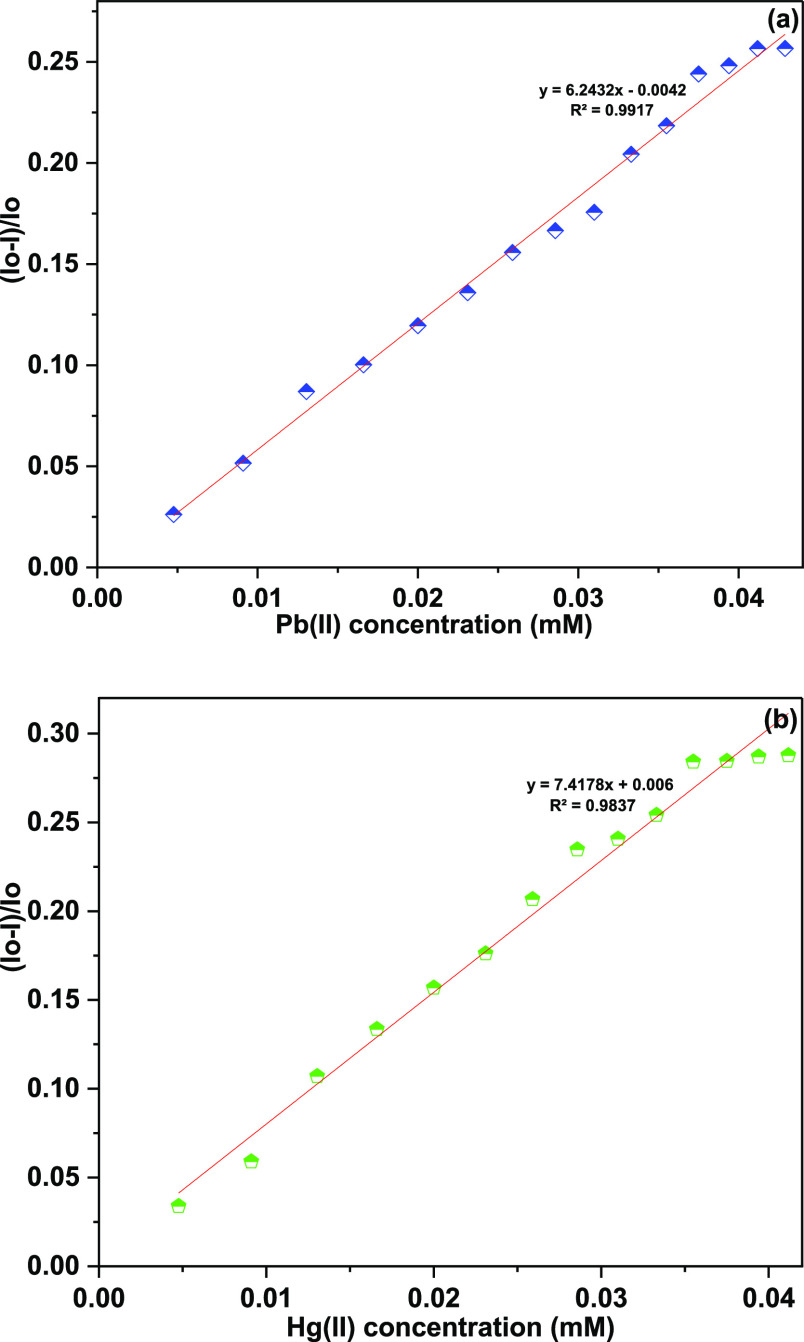
Correlation plot signifying the relative change in the fluorescence emission intensity of TCT (*I*_0_ – *I*)/*I*_0_ vs metal ion concentration [(a) Pb(II) ions and (b) Hg(II) ions]; *I*_0_ = initial fluorescence emission of TCT (in the absence of metal ions) and *I* = fluorescence emission of TCT in the presence of metal ions.

**Figure 7:**
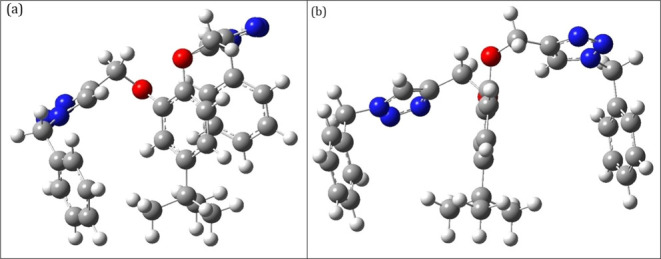
Optimized structure of TCT using DFT with the hybrid density functional (B3LYP)/6311G++(d,p) basis set of theory via the Gaussian 09 package: (a) front view and (b) side view.

The binding ability of the probe TCT was also established through fluorescence spectroscopic titrations. The probe on excitation at a frequency of 280 nm (λ_ex_) exhibited emission maximum (λ_ems_) with an intense band at 340 nm and an additional band at 653 nm of lower intensity. The peak at 340 nm corresponds to the monomer emission, whereas the peak at 653 nm is attributable to the excimer formation as supported by the DFT-optimized structure of the synthesized probe (Figure 7), wherein π–π stacking of the benzyl groups gives rise to the excimer.^39^ The relative intensity ratio of the monomer and excimer band for free TCT (M_339_/E_653_) was 2.45, which increased to 2.53 on addition of either of the metal ions, attributing to the formation of the TCT–metal complex. On addition of Pb(II) and Hg(II) ions in separate titrations to TCT (50 μM concentration), between the concentration range of 4 and 40 μM, an enhancement of fluorescence emission at both 340 nm and 653 nm was observed with an isosbestic point at about 630 nm, as represented in Figure 5. The inset for both the plots shows the relative change in fluorescence emission (*I*/*I*_0_) versus metal ion concentration, where *I* = fluorescence emission intensity of TCT with addition of metal ions and *I*_0_ = fluorescence intensity of TCT in the absence of metal ions. Furthermore, analysis of the correlation plot (*I*_o_ – *I*_n_)/*I*_o_ versus Pb(II) concentration (Figure 6a) revealed the limit of detection and the limit of quantification (LoQ) of the probe to be 8.6 and 28.7 μM, respectively, whereas the Job plot confirmed the metal-to-ligand binding ratio to be 2:1 (Table 1). Similarly, correlation plot for Hg(II) (Figure 6b) presented the LoD and the LoQ of the probe to be 11 and 38 μM, respectively, and the Job plot confirmed the metal-to-ligand binding ratio of 2:1 (Table 1).

**Table 1:** LoD, LoQ, and Stoichiometric Values of TCT on Binding with Pb(II) and Hg(II)

entry	metal ion	LoD (μM)	LoQ (μM)	stoichiometry
TCT	Pb(II)	8.6	28.7	2:1
TCT	Hg(II)	11	38	2:1

### 3.5 TCT–Metal Complex Synthesis and Confirmation of Metal–Ligand Binding

The metal–ligand complex was synthesized by refluxing the solution of metal and ligand (in a molar ratio of 2:1[L/M]) for 4 h in CH_3_CN. Color change from pale yellow to dark yellow was observed, thereby indicating the formation of the complex. The solid product was obtained after filtering the solution and removing the solvent under vacuum. The ^1^H NMR and IR spectra of the metal–ligand complex were obtained as represented in the Supporting Information (Figures S16 and S17, respectively). Comparing the ^1^H NMR spectrum of probe TCT with that of the TCT–metal complex revealed a significant downfield shift for the triazole proton peaks at δ = 7.57 and 7.59 to δ = 7.65 and 7.69, respectively, while the remaining peaks exhibited no shifting. Also, disappearance of the peak at 3134 cm^–1^ in the IR spectrum of TCT–metal complex indicated the transfer of electron density from the triazole ring to the metal–N bond. Therefore, the ^1^H NMR and IR spectra evidenced the binding of the metal ions to the ligand molecules.

## 4 Computational Studies

### 4.1 Structure Optimization via DFT

**Figure 8:**
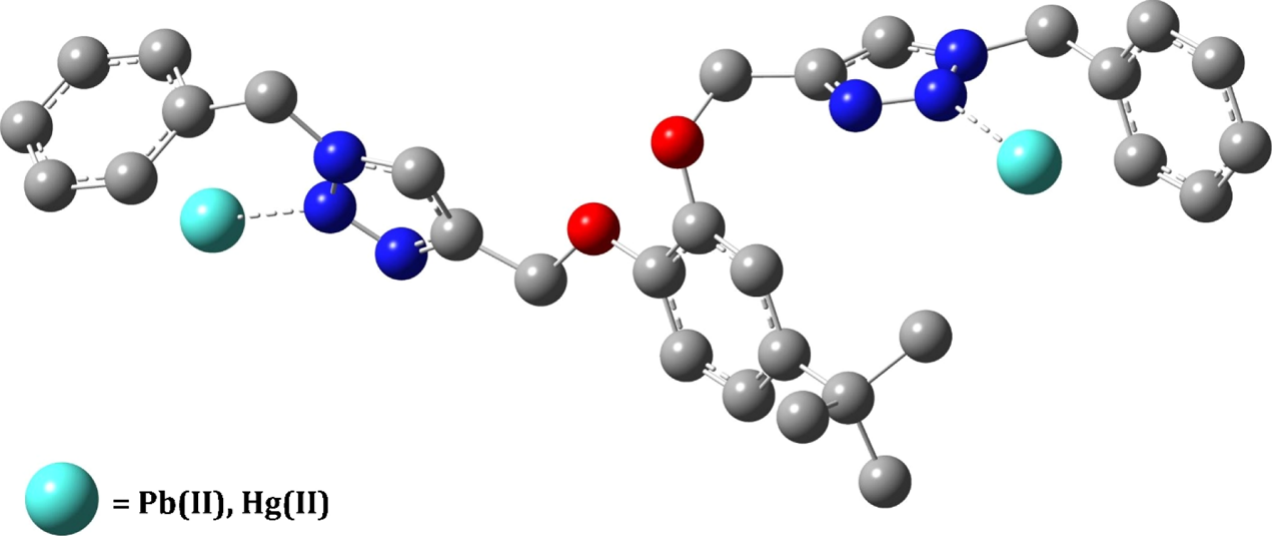
Optimized structure of the TCT–metal complex using DFT with the hybrid density functional (B3LYP)/LANL2DZ via the Gaussian 09 package (H-atoms have been omitted for clarity).

**Figure 9:**
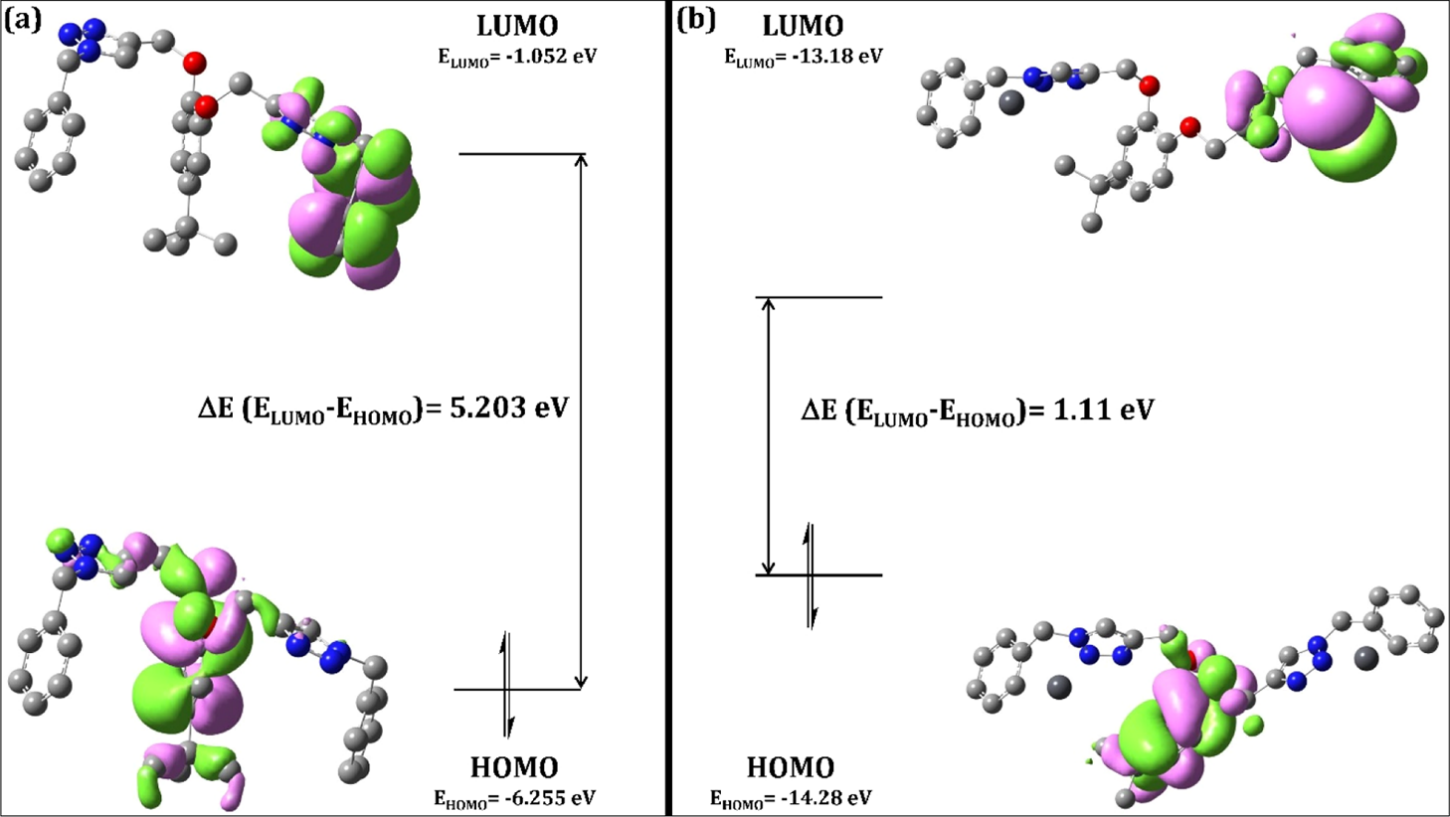
Depiction of HOMO–LUMO density plot of (a) TCT and (b) TCT–metal complex with their corresponding calculated Δ*E* (*E*_LUMO_ – *E*_HOMO_) values (H-atoms have been omitted for clarity).

DFT has become a computational tool of utmost importance, which provides structural insights, especially about large molecules with extended conjugation by applying quantum mechanical considerations. The binding approach of the probe TCT toward the metal ions was also explored via DFT calculations. The energy-minimized structure of TCT and TCT–metal complex was obtained after a computational investigation using DFT with the B3LYP hybrid functional and the 6311G++(d,p) basis set for TCT and the B3LYP/LANL2DZ set for the TCT–metal complex via the Gaussian 09 package.^40^ The optimized structure of TCT is illustrated in Figure 7 (Cartesian coordinates are provided in the Supporting Information, Table S1) and that of the TCT–metal complex is depicted in Figure 8, wherein the metal ion is shown to get attached to one of the N atoms of the 1,2,3-triazole moiety, as confirmed through the ^1^H NMR spectrum of the metal–ligand complex. Furthermore, the density plot of the HOMO and the LUMO over the molecular structure with their energy difference for both TCT and TCT–metal complex is represented in Figure 9a,b, respectively, wherein the HOMO electron density is mostly delocalized over the parent 4-*tert*-butylcatechol ring, whereas the electron density for LUMO is delocalized over one of the arms attached to the 4-*tert*-butylcatechol ring bearing the 1,2,3-triazole moiety and the aromatic six-membered ring. The lower Δ*E* value for the TCT–metal complex (1.11 eV) as compared to TCT (5.203 eV) is indicative of extra stability of the metal–ligand complex.

### 4.2 Molecular Docking Analysis

**Figure 10:**
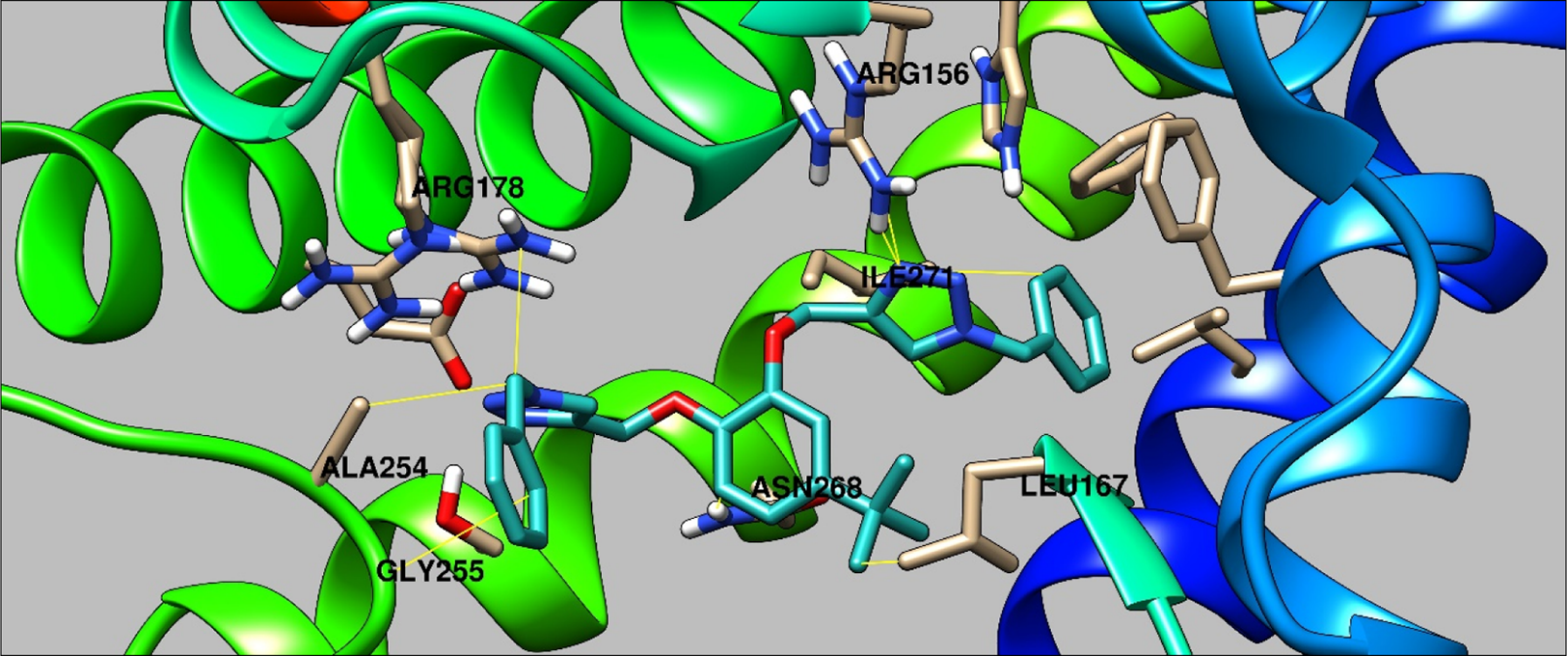
Pictorial depiction of binding of TCT with the different amino acid residues in the protein pocket of leukotriene b4. (Visualized using the UCSF Chimera).^44^

1,2,3-Triazole derivatives have been reported in the literature to possess certain pharmacophore properties such as antibacterial, antifungal, anticancer, anti-inflammatory agents, and so forth.^41^ The synthesized TCT was explored for its potential to inhibit leukotriene synthesis, a proinflammatory mediator^42^ in the body, and hence subsequently docked against the human leukotriene b4 protein through AutoDock Vina.^43^ The probe exhibited a binding affinity of −10.2 kcal/mol for the protein, and the binding interactions of the probe with the protein are represented in Figure 10, wherein it is specified that the probe is incorporated in the active site of the probe via interaction of its different atoms of both the arms and the parent ring with various amino acid residues, namely, ARG156, LEU167, ARG178, ALA254, GLY255, ASN268, and ILE271.

## 5 Probable Binding Mode

**Figure 11:**
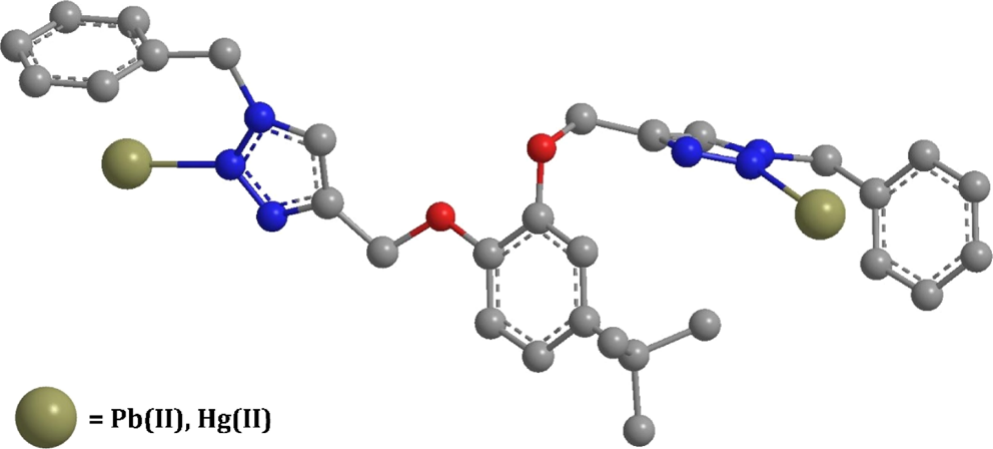
Plausible binding mode of TCT with Pb(II) and Hg(II).

The HSAB concept categorizes Pb(II) as a borderline acid and Hg(II) as a soft acid.^45^ Both the metal ions are capable of bond formation with groups having lone pair bearing atoms such as N, O, and S. TCT is equipped with N atoms bearing lone pairs on the 1,2,3-triazole moiety, thereby enabling it to trap the incoming metal ions by providing suitable binding sites. The Job plot of the probe with both Pb(II) and Hg(II) is in well agreement with the formation of a 2:1 metal–ligand complex. Furthermore, the ^1^H NMR and IR analysis of the TCT–metal complex collaborated with the DFT calculations suggested the binding of the metal ions with the N atoms of the 1,2,3-triazole moiety. On the basis of all the above considerations, a tentative binding mode of the receptor with the metal ions is illustrated in Figure 11, wherein it is speculated that each arm comprising the triazole moiety adjusts in such a way that the incoming metal ion gets bound to the N atom of the triazole ring on either side of the molecule.

## 6 Conclusions

The constant accumulation of toxic heavy metal ions, especially lead and mercury in and around living systems, has posed a serious threat owing to their harmful effects and complications thereof. As a result, keeping a check on the presence of heavy metal ions in the environment and adopting time-efficient and relatively inexpensive methodologies for the detection of the same has become the most pressing need at the present time. 1,2,3-Triazole-annexed ensembles, synthesized via the CuAAC methodology, have been extensively explored by researchers for ion sensing properties and testified to be custom-made to serve the needful purpose. Considering this approach, a 1,2,3-triazole-tethered chemosensor probe starting from 4-*tert*-butylcatechol was synthesized following the CuAAC “click” pathway, which when subjected to an ion sensing procedure exhibited selective recognition for Pb(II) and Hg(II) ions, both of which are well known to induce grave damages in the living systems on exceeding their permissible limits. The ion detection behavior of the synthesized probe was established using UV–vis and fluorescence spectroscopy, wherein significant deflections were observed in the absorption as well as the emission spectrum of the probe on the addition of metal ions. Additionally, the energy-minimized structure of the probe was determined via DFT studies to get an understanding of the spatial arrangement of the various groups, and afterward, the anti-inflammatory characteristic of the probe was explored by docking it with the proinflammatory human leukotriene b4 protein using AutoDock Vina..

## Supporting Information Available

IR, ^1^H NMR, ^13^C NMR, and mass spectra of TCT and its precursors; UV–vis spectra of pure TCT, in the presence of various metal ions and in coexistence of Pb(II) and Hg(II); time-dependent and temperature-dependent absorption spectra of TCT–metal complexes; IR and ^1^H NMR spectra of TCT–metal complex; and Cartesian coordinates of TCT (PDF)
