# Supplementary material for: CuAAC-Derived Selective Fluorescent Probe as a Recognition Agent for Pb(II) and Hg(II): DFT and Docking Studies
Source: ACS Omega. 2022 Oct 20;7(43):39159–68. doi: 10.1021/acsomega.2c05050 (PMC9631732; doi:10.1021/acsomega.2c05050)
Supplement: Supplementary file 1 — ao2c05050_si_001.pdf [file ao2c05050_si_001.pdf]

CuAAC derived selective fluorescent probe as recognition agent for Pb(II) and Hg(II), DFT and docking studies

Gurleen Singh,[a] Nancy George,[a] Riddima Singh,[a] Gurjaspreet Singh,[b] Jashan Deep Kaur,[b] Gurpreet Kaur,[c] Harminder Singh,[a,#] Jandeep Singh,[a,*]

[a] School of Chemical Engineering and Physical Sciences, Lovely Professional University, Phagwara–144411 (Punjab)

[b] Department of Chemistry and Centre of Advanced Studies in Chemistry, Panjab University, Chandigarh–160014

[c] Department of Chemistry, GGN Khalsa College, Ludhiana–141001 (Punjab)

#,* Corresponding Authors

Contact details:

***singhjandeep@gmail.com**

Supporting Information

Contents:

Figure S1: IR spectrum of alkyne **2**

Figure S2: ^{1}H NMR spectrum of alkyne **2**

Figure S3: ^{13}C NMR spectrum of alkyne **2**

Figure S4: IR spectrum of benzyl azide

Figure S5: ^{1}H NMR spectrum of benzyl azide

Figure S6: ^{13}C NMR spectrum of benzyl azide

Figure S7: IR spectrum of probe **TCT**

Figure S8: ^{1}H NMR spectrum of probe **TCT**

Figure S9: ^{13}C NMR spectrum of probe **TCT**

Figure S10: Mass spectrum of probe **TCT**

Figure S11: UV-Vis spectra of triazole probe **TCT** (0.4 mM) in CH_3CN/H_2O (4:1) depicting absorption maxima at λ_{max}= 277 nm

Figure S12: UV-Vis spectra of triazole probe **TCT** (0.4 mM) in CH_3CN/H_2O (4:1) signifying the recognition of Hg(II) among various metal ions present in equimolar concentration

Figure S13: UV-Vis spectra of triazole probe **TCT** (0.4 mM) in CH_3CN/H_2O (4:1) demonstrating the selective detection of Hg(II) over Pb(II) from an equimolar concentration solution of both the ions

Figure S14: Time dependent spectra of **TCT**-metal complex solution displaying the trend in the absorption intensity at λ_{max} = 277 nm with the passage of time: (a) **TCT**-Pb(II) complex (b) **TCT**-Hg(II) complex

Figure S15: Changes exhibited in the absorption spectrum of **TCT**-metal complex solution on exposure to different temperature: (a) **TCT**-Pb(II) and (b) **TCT**-Hg(II)

Figure S16: ^{1}H NMR of TCT-metal complex

Figure S17: IR spectrum of TCT-metal complex

Table S1: Cartesian co-ordinates of probe **TCT**

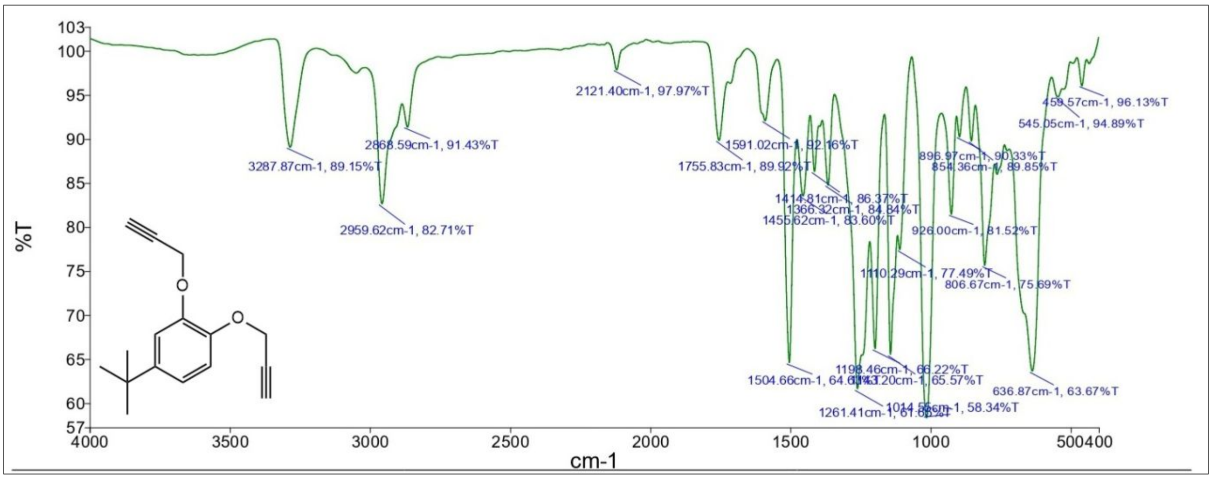

Figure S1. IR spectrum of alkyne **2**

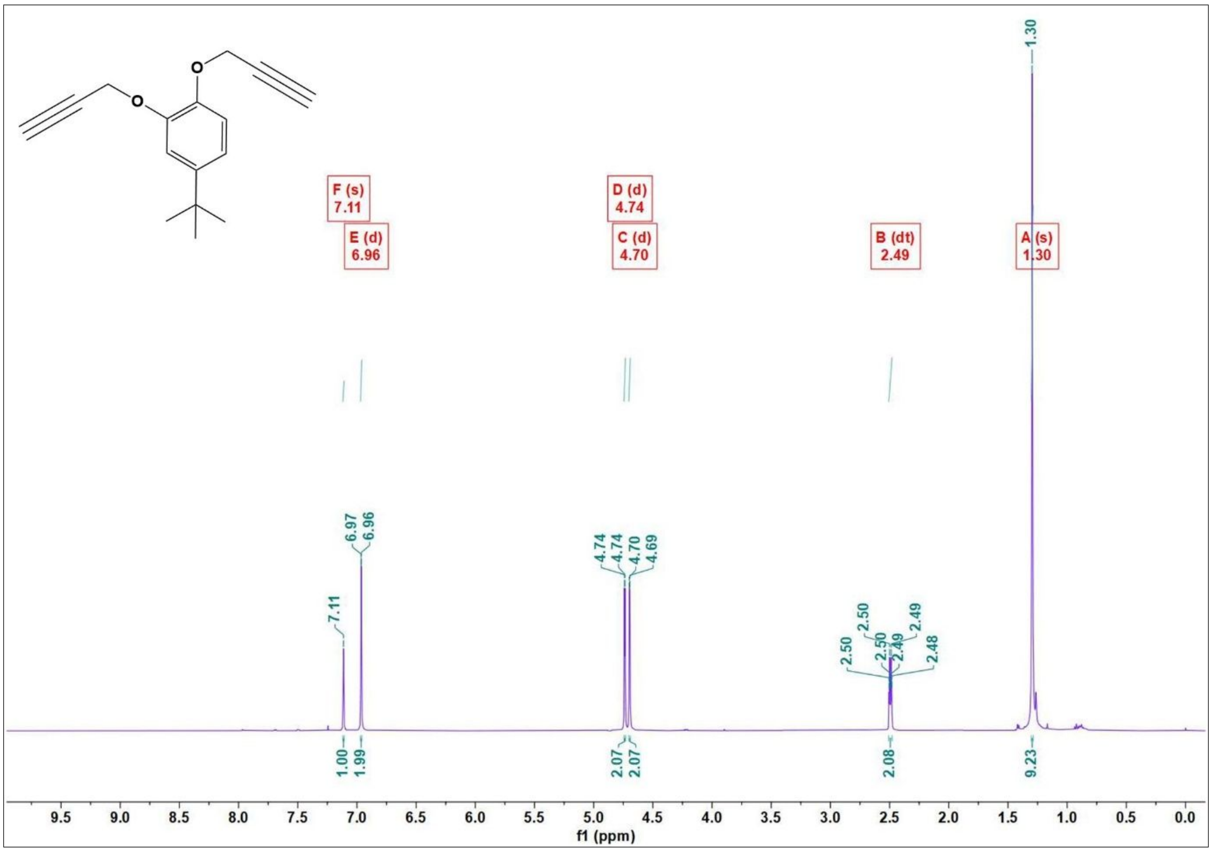

Figure S2. ^{1}H NMR spectrum of alkyne **2**

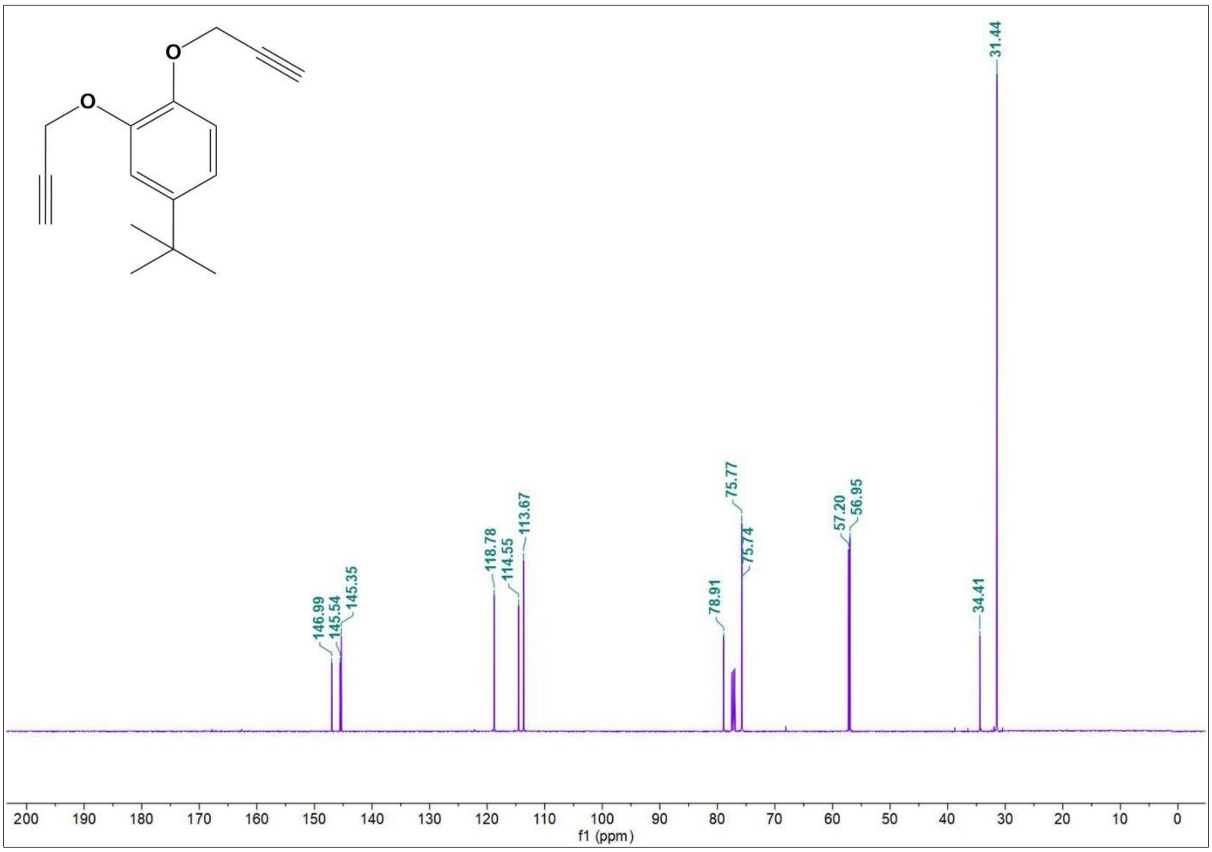

Figure S3. ^{13}C NMR spectrum of alkyne **2**

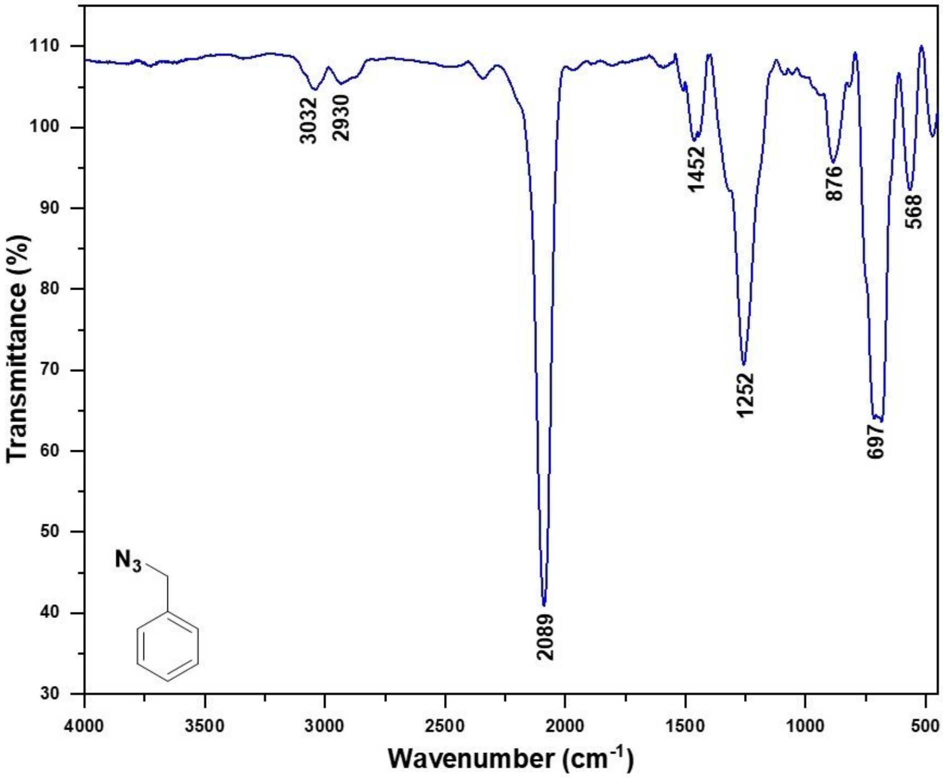

Figure S4. IR spectrum of benzyl azide

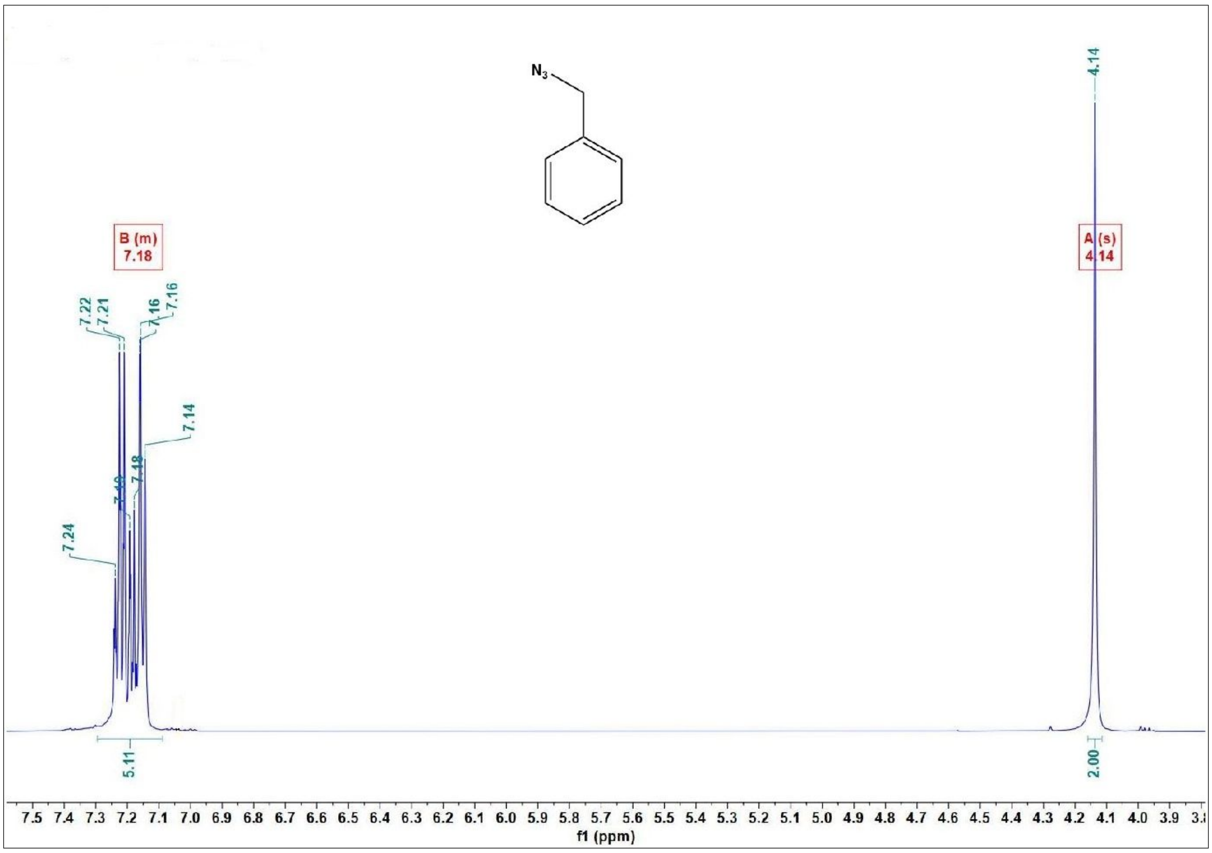

Figure S5. ^{1}H NMR spectrum of benzyl azide

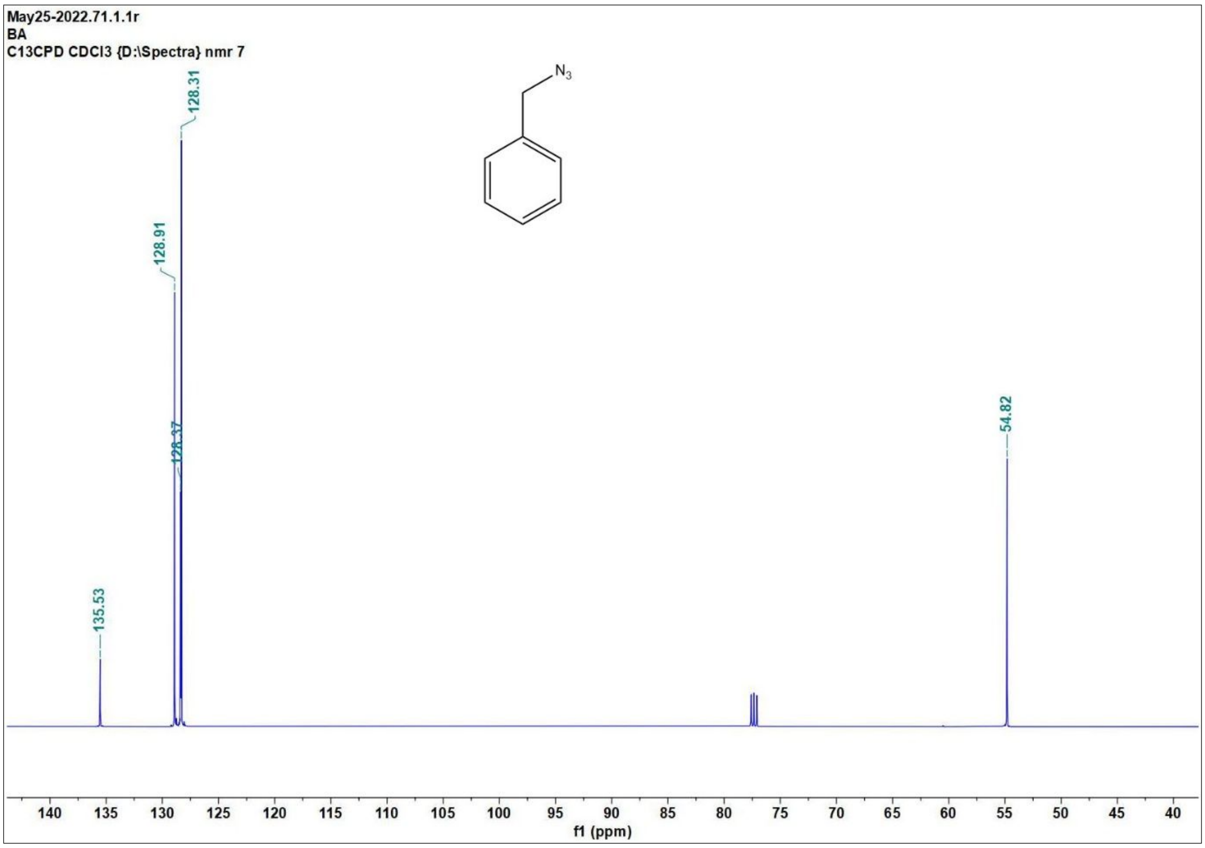

Figure S6. ^{13}C NMR spectrum of benzyl azide

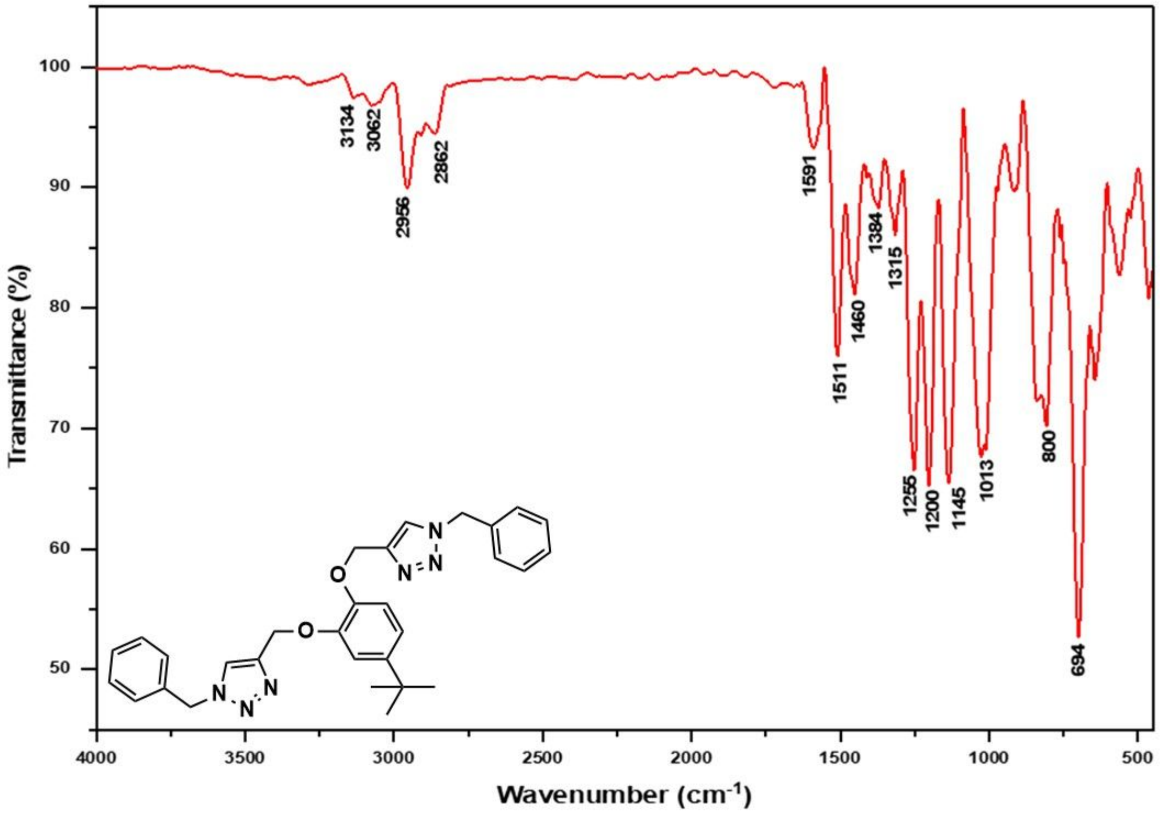

Figure S7. IR spectrum of probe **TCT**

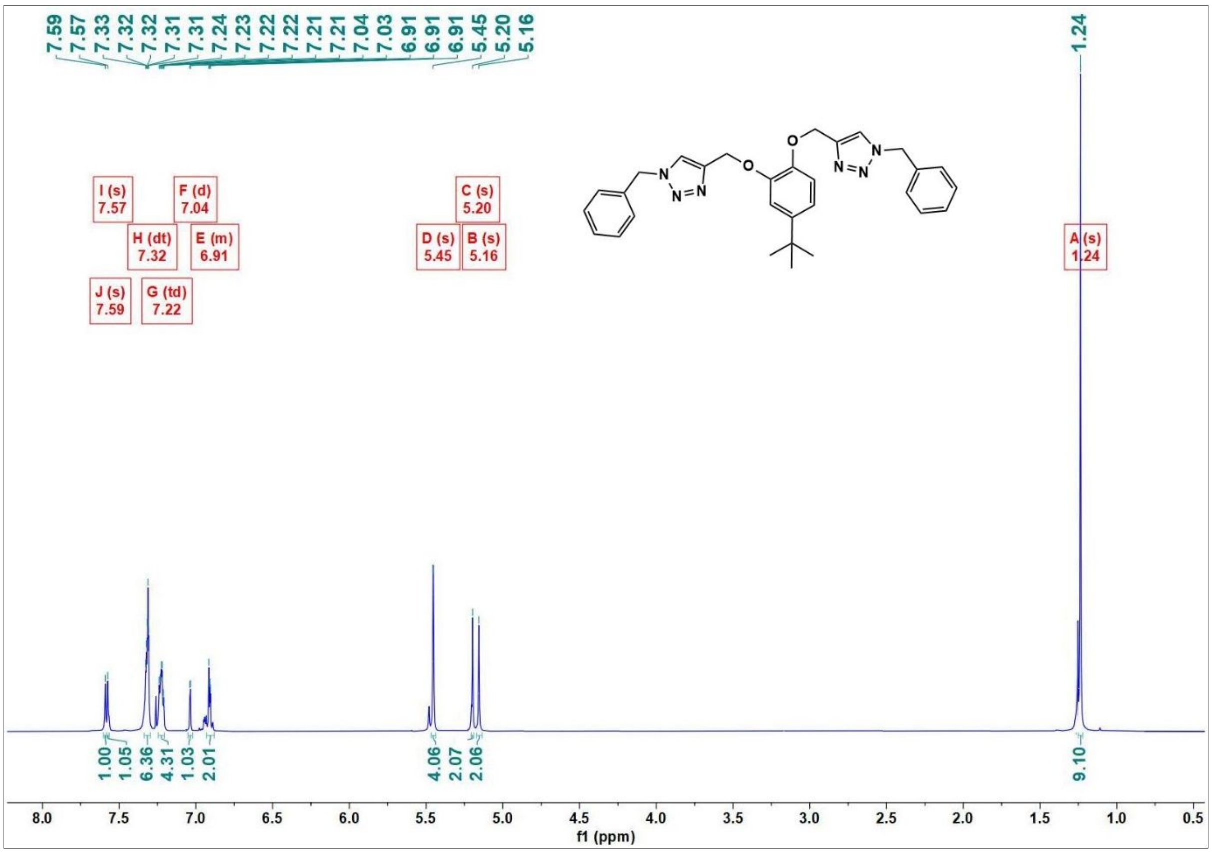

Figure S8. ^{1}H NMR spectrum of probe **TCT**

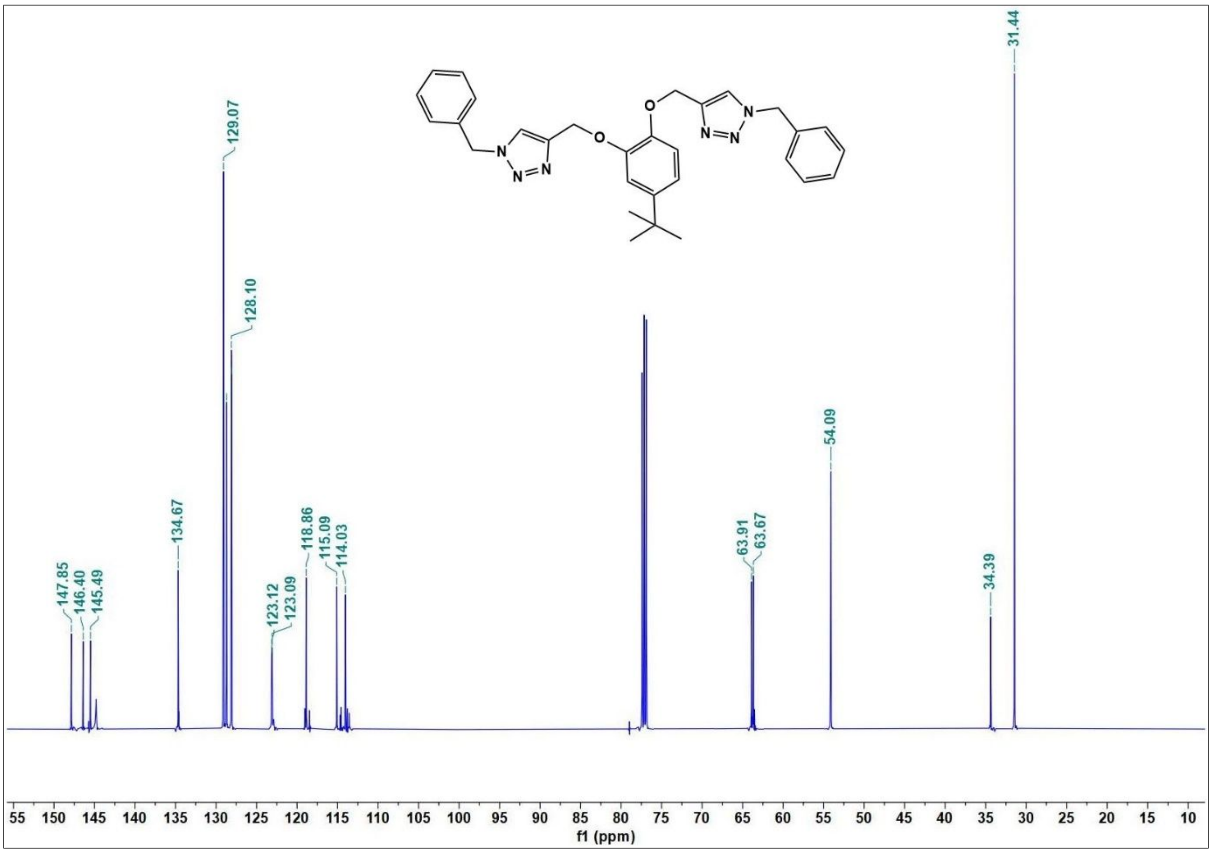

Figure S9. ^{13}C NMR spectrum of probe **TCT**

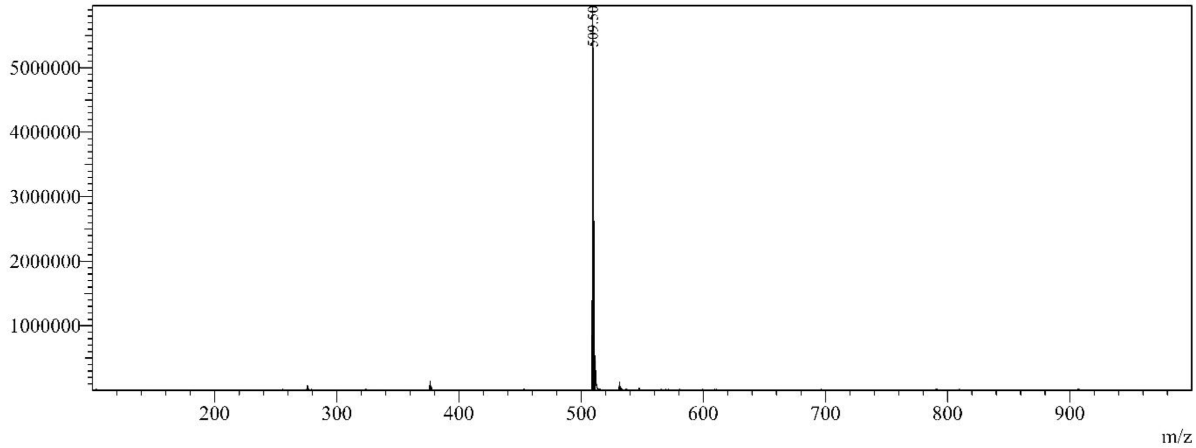

Figure S10. Mass spectrum of probe **TCT**

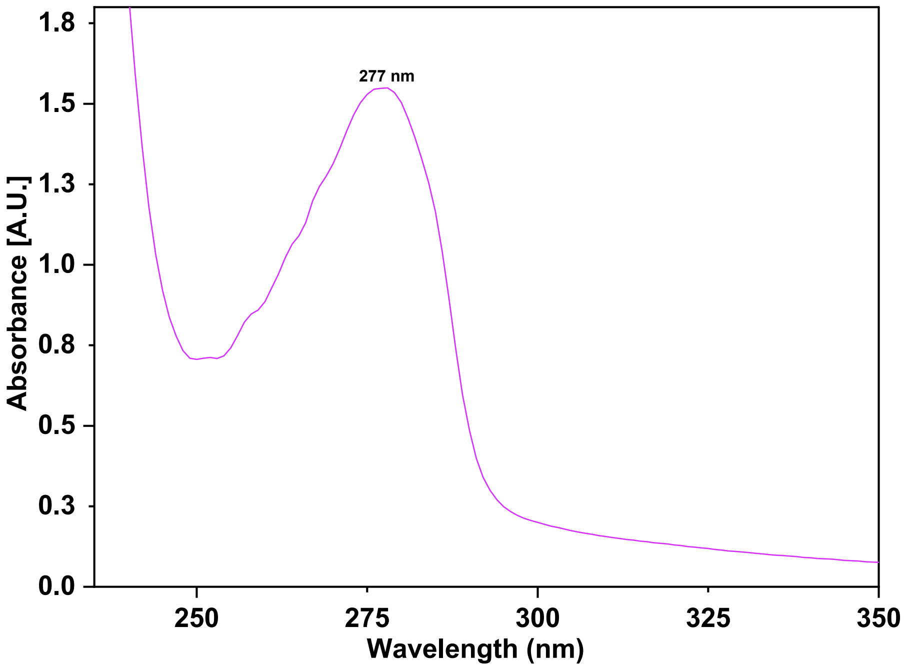

Figure S11. UV-Vis spectra of triazole probe **TCT** (0.4 mM) in CH_3CN/H_2O (4:1) depicting absorption maxima at λ_{max}= 277 nm

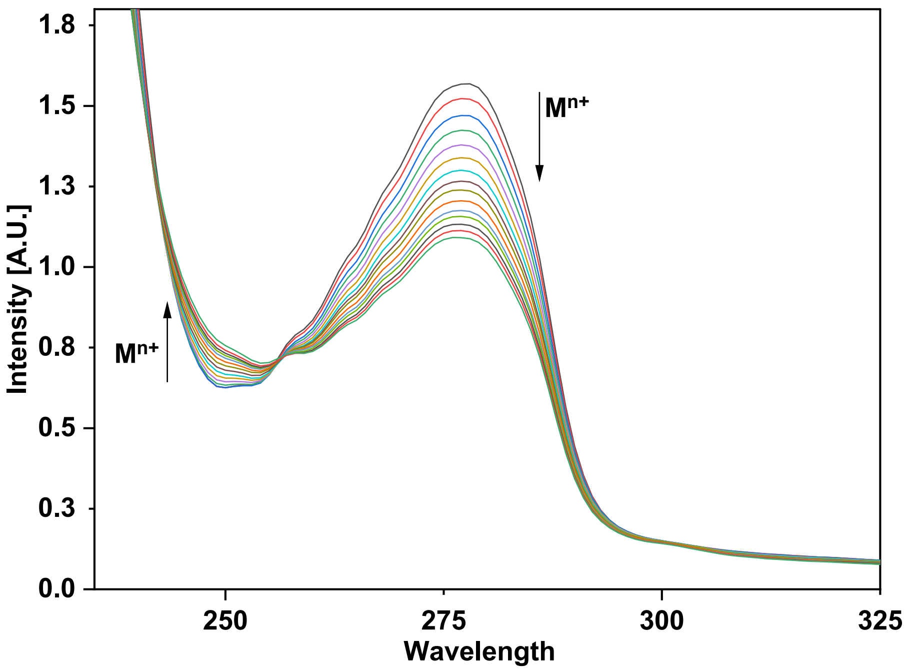

Figure S12. UV-Vis spectra of triazole probe **TCT** (0.4 mM) in CH_3CN/H_2O (4:1) signifying the recognition of Hg(II) among various metal ions present in equimolar concentration

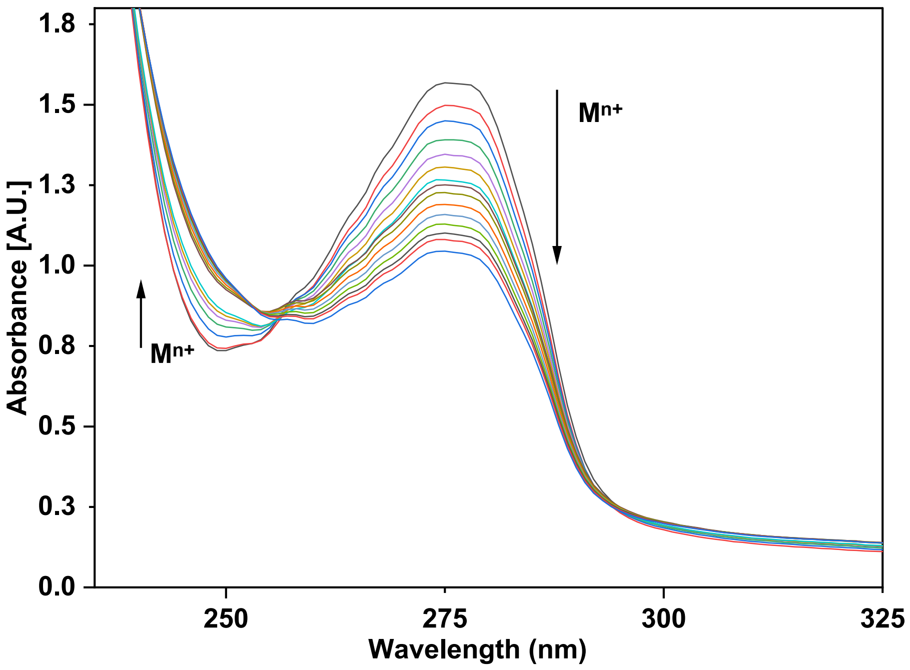

Figure S13. UV-Vis spectra of triazole probe **TCT** (0.4 mM) in CH_3CN/H_2O (4:1) demonstrating the selective detection of Hg(II) over Pb(II) from an equimolar concentration solution of both the ions

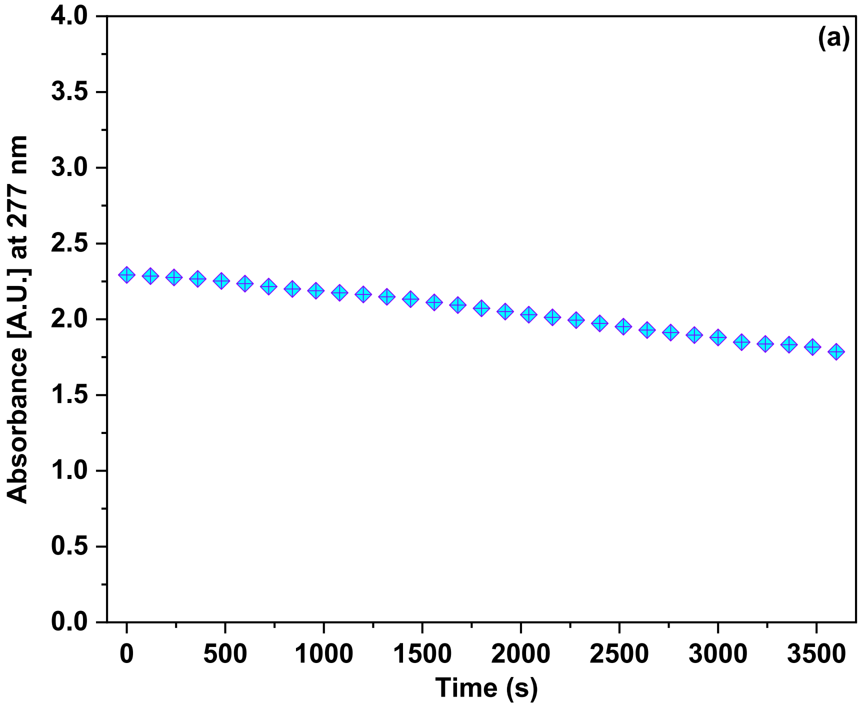

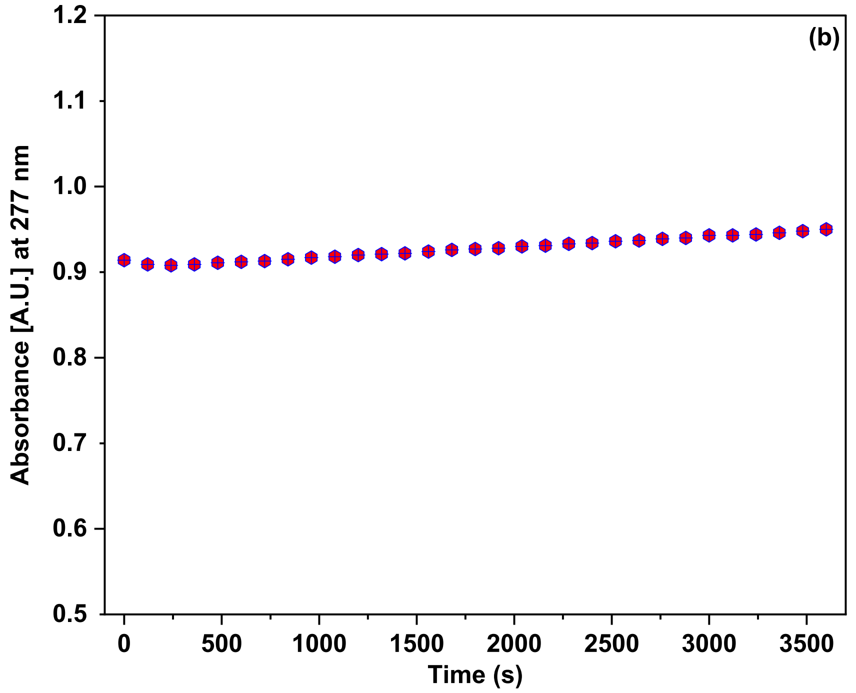

Figure S14. Time dependent spectra of **TCT**-metal complex solution displaying the trend in the absorption intensity at λ_{max} = 277 nm with the passage of time: (a) **TCT**-Pb(II) complex (b) **TCT**-Hg(II) complex

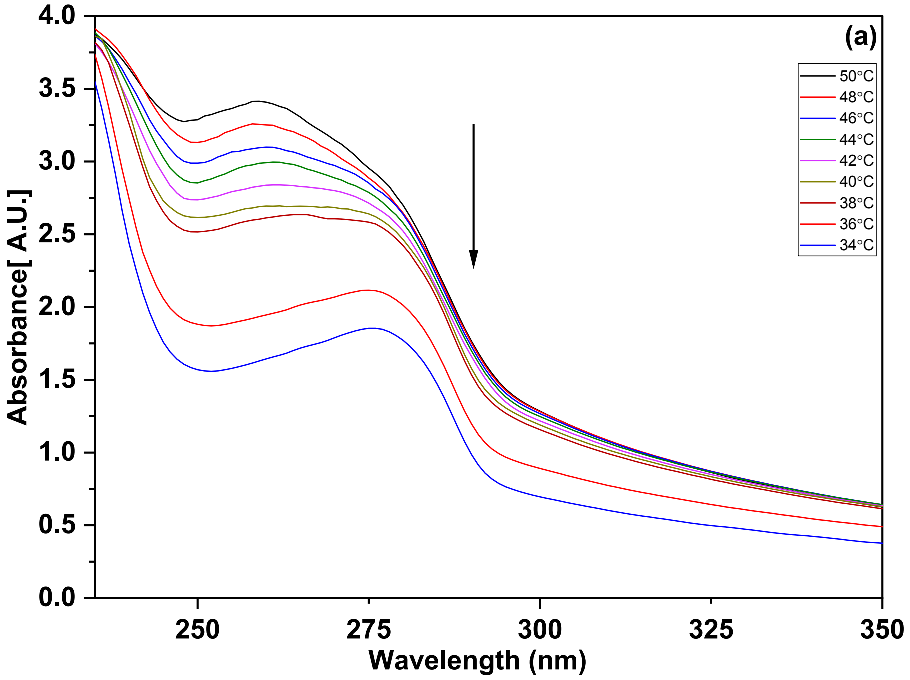

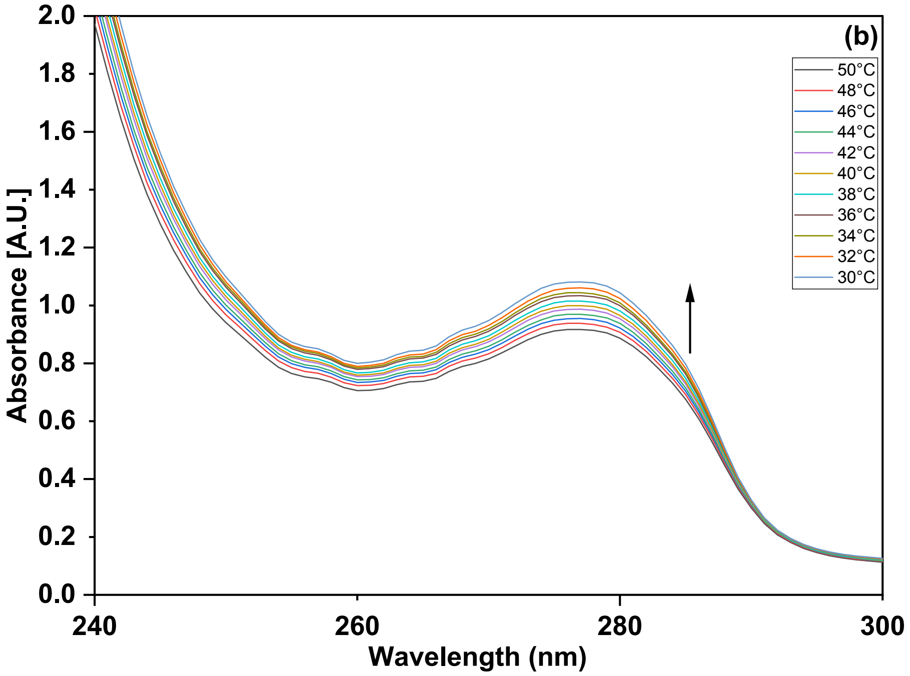

Figure S15. Changes exhibited in the absorption spectrum of **TCT**-metal complex solution on exposure to different temperature: (a) **TCT**-Pb(II) and (b) **TCT**-Hg(II)

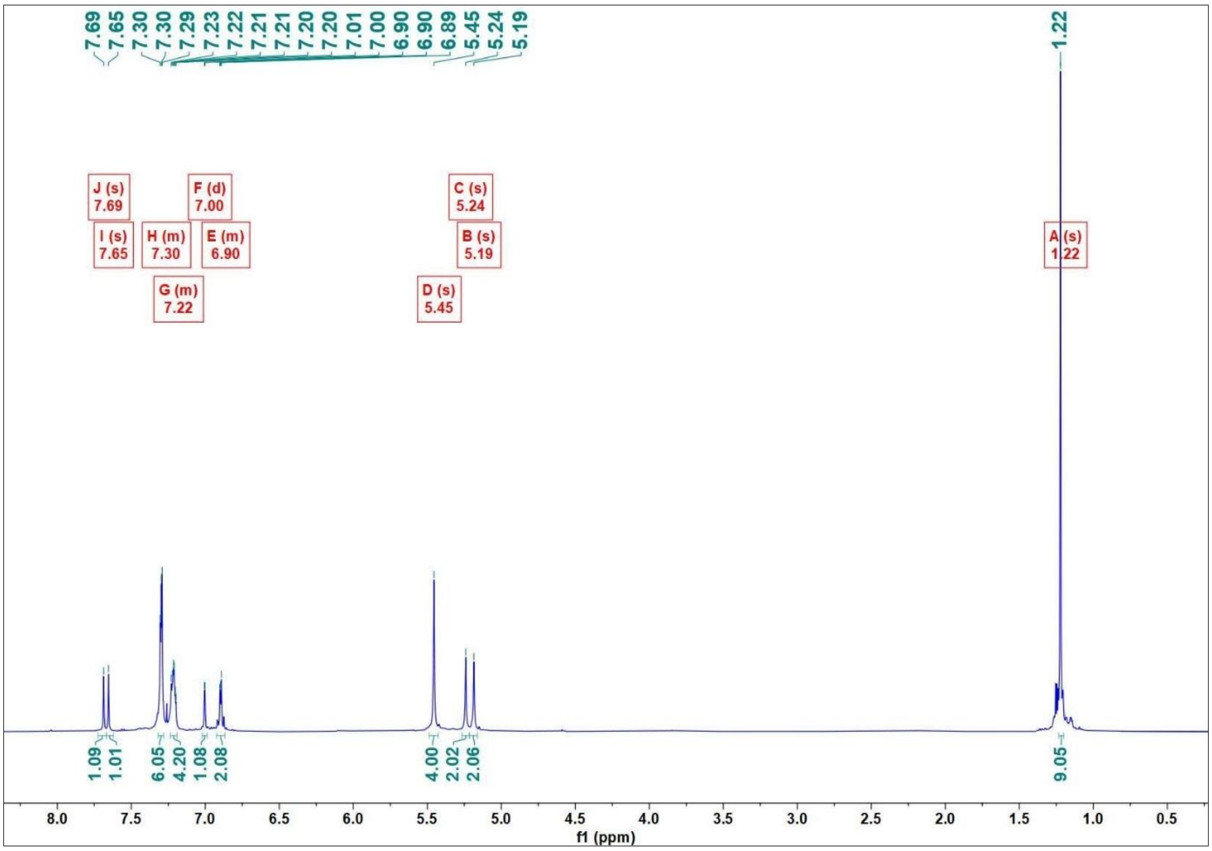

Figure S16. ^{1}H NMR spectrum of **TCT**-metal complex

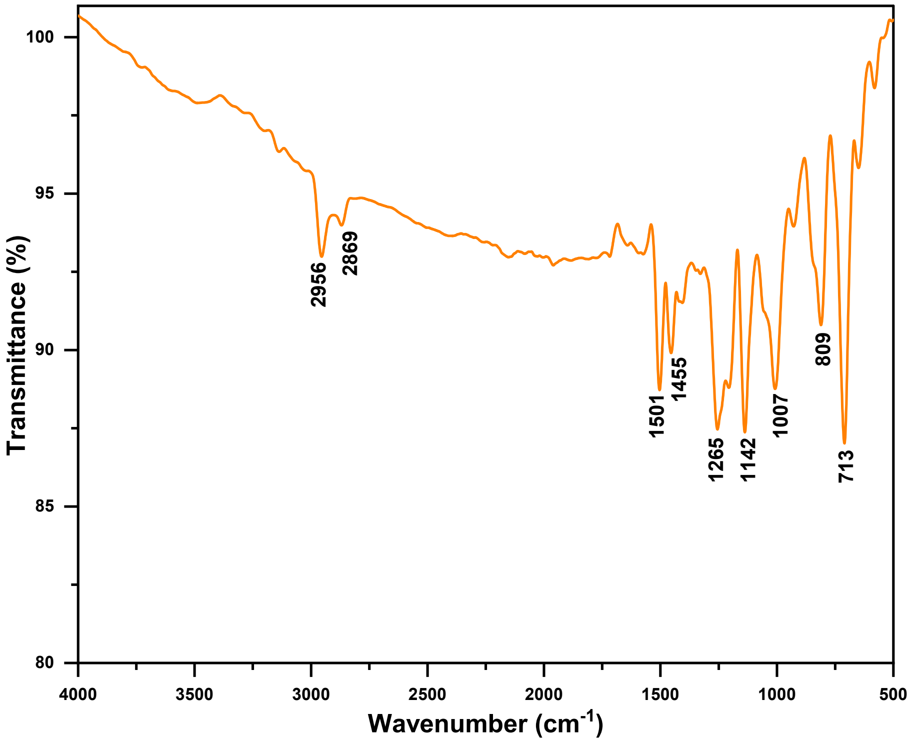

Figure S17. IR spectrum of **TCT**-metal complex

O	-0.78826	-2.26617	1.62022
C	0.87184	3.15045	0.62262
C	0.44825	1.70513	0.93823
C	2.19988	3.14372	-0.16822
C	1.07812	3.98816	1.89779
C	-0.22711	3.82847	-0.22885
C	0.25848	0.79149	-0.10725
C	0.22465	1.24121	2.23788
C	-0.15808	-0.51628	0.11709
C	-0.1852	-0.06994	2.48093
C	-0.39915	-0.95446	1.42976
H	2.50815	4.16887	-0.39744
H	2.9974	2.66844	0.40886
H	2.10745	2.6054	-1.11412
H	1.85991	3.56916	2.53752
H	1.38374	5.00175	1.62412
H	0.15987	4.06982	2.4858
H	0.0576	4.8586	-0.46583

H	-0.39088	3.30112	-1.17139
H	-1.17875	3.85242	0.30902
H	0.41133	1.07891	-1.1404
H	0.36608	1.89546	3.0875
H	-0.34883	-0.39935	3.50042
C	-1.95003	-2.51065	2.42204
C	0.37024	-2.51599	-1.14022
H	-0.08272	-3.01405	-1.99707
H	0.25932	-3.14488	-0.25588
H	-1.92832	-3.5843	2.62116
H	-1.88996	-1.99685	3.38578
O	-0.42217	-1.31276	-0.97643
C	-3.21973	-2.1273	1.72279
C	-3.4501	-1.82561	0.39954
H	-2.79084	-1.74111	-0.44866
C	1.81106	-2.25738	-1.42988
C	2.89808	-2.26545	-0.5815
H	2.98928	-2.43016	0.4789
N	2.26566	-1.99001	-2.6901
N	3.55528	-1.83986	-2.66096
N	3.9595	-2.00663	-1.38055
C	5.38154	-1.91024	-1.04344
H	5.63941	-2.76338	-0.41276
H	5.90654	-2.0227	-1.99325
C	5.76536	-0.61261	-0.36286
C	5.6765	0.60072	-1.05391
C	6.23886	-0.61429	0.95044
C	6.05382	1.78981	-0.43731
H	5.30822	0.61011	-2.07435
C	6.61916	0.57689	1.56918
H	6.31755	-1.55099	1.49356
C	6.5272	1.78067	0.87567
H	5.98259	2.72445	-0.98194
H	6.98652	0.56182	2.58908

H	6.82378	2.70771	1.35314
N	-4.40325	-2.06105	2.39284
N	-5.3502	-1.74119	1.55644
N	-4.78574	-1.59485	0.34109
C	-5.60133	-1.17552	-0.79952
H	-6.63037	-1.40207	-0.51554
H	-5.34005	-1.80249	-1.65407
C	-5.44418	0.29094	-1.14878
C	-5.79059	1.27907	-0.2205
C	-4.97308	0.67307	-2.40576
C	-5.66491	2.62564	-0.54818
H	-6.15301	0.98871	0.75999
C	-4.85033	2.02285	-2.73661
H	-4.69879	-0.08553	-3.132
C	-5.19574	3.00095	-1.80806
H	-5.93615	3.38365	0.17808
H	-4.48216	2.30639	-3.71612
H	-5.10022	4.05055	-2.06234

Table S1. Cartesian co-ordinates of probe **TCT**
